# Aqueous Carbon Capture Using Guanidinium-Functionalized Hollow Fiber Sorbent Contactors

**DOI:** 10.1021/jacsau.5c01639

**Published:** 2026-03-30

**Authors:** Mary K. Danielson, Nicholas Gregorich, Mary H. Irwin, Cyril Pepple, Anton S. Pozdeev, Alexander S. Ivanov, Joshua Damron, Zachary Coin, Tarryn Trick, Tomonori Saito, Ramesh Bhave, João Marreiros, Ryan P. Lively, Syed Z. Islam, Md Anisur Rahman

**Affiliations:** † Chemical Sciences Division, 6146 Oak Ridge National Laboratory, Oak Ridge, Tennessee 37831, United States; ‡ Circular Bioeconomy Systems Convergent Research Initiative, University of Tennessee Oak Ridge Innovation Institute, Knoxville, Tennessee 37996, United States; § Materials Science and Engineering, 4292 University of Tennessee, Knoxville, Tennessee 37996, United States; ∥ School of Chemical and Biomolecular Engineering, 1372 Georgia Institute of Technology, Atlanta, Georgia 30332, United States; ⊥ Department of Chemical & Biomolecular Engineering, Vanderbilt University, Nashville, Tennessee 37235, United States; # Department of Nuclear Engineering, University of Tennessee, Knoxville, Tennessee 37996, United States; ¶ Department of Chemistry, 7548 Washington University in St. Louis, St. Louis, Missouri 63130, United States; ∇ Bredesen Center for Interdisciplinary Research and Graduate Education, University of Tennessee, Knoxville, Tennessee 37996, United States

**Keywords:** polymer sorbents, fiber contactors, carbon capture, pH swing, ion exchange

## Abstract

As part of the growing suite of technologies aimed at combatting rising temperatures, negative emissions technologies have become a powerful tool in the global effort to minimize the consequences of human-induced climate change. Among these, carbon removal from aqueous sources, which contain much higher carbon concentrations than the atmosphere, remains largely unexplored. Indeed, developing robust and efficient carbon capture materials for usage in complex aqueous environments remains a significant challenge. Here, we explore the potential of functionalizing polyvinylidene fluoride (PVDF) hollow fiber contactors grafted with a guanidinium-derived polymer sorbent for carbon removal from aqueous sources, including saline waters. Computational screening against amine-based analogs is utilized to identify guanidinium as a promising motif for bicarbonate (HCO_3_^–^) ions binding. To leverage this finding, synthesis of a guanidinium polymer and subsequent covalent grafting onto PVDF hollow fibers is employed to structured polymer–sorbent–grafted hollow fiber contactors. Our prototype achieves an initial HCO_3_^–^ removal of 34% with an increase to 98% after four cycles. The functionalized fibers demonstrate aqueous stability over 13 adsorption/desorption cycles in model NaHCO_3_ solutions where regeneration is facilitated by a mild pH swing. Importantly, the system maintains selective performance in the presence of competitive chloride ions over multiple cycles; carbon removal remained above 10% even at high (10:1) NaCl/NaHCO_3_ ratios. These findings demonstrate the feasibility of sorbent-based aqueous carbon removal and highlight its potential as a promising approach for negative emissions.

## Graphical abstract

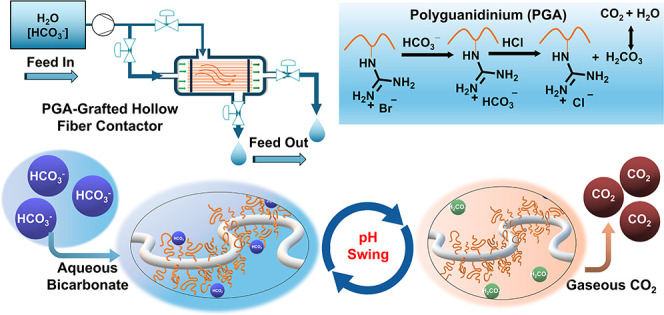

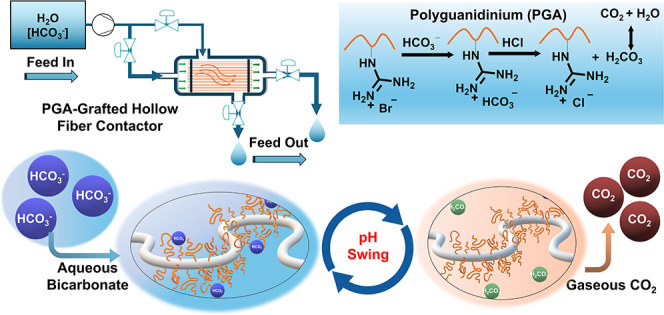

## Introduction

Climate change is one of the most critical challenges of our time, with profound implications for ecosystems, human societies, and the global economy. According to the Intergovernmental Panel on Climate Change (IPCC), to reduce the risk of catastrophic damage, between 100 and 1000 billion tons of CO_2_ will need to be removed from the atmosphere by 2100, with 5–15 billion tons being removed by 2050.^1,2^ Currently, most negative emissions technologies (NETs) prioritize carbon removal from gaseous feedstocks (e.g., air and flue gas), while the potential of carbon capture from aqueous environments remains relatively unexplored. The ocean alone absorbs 25%–31% of the world’s CO_2_, with a 140% higher storage of CO_2_ as inorganic carbon than the atmosphere.^3^ In ocean water, the inorganic carbon consists of salts comprised of bicarbonate (HCO_3_^–^) (∼88%), carbonate (CO_3_^2–^) (11%), and, to a lesser extent, dissolved gaseous CO_2_ (1%). Though the ocean accounts for most of the dissolved inorganic carbon (DIC) given its surface area, the DIC concentration of the ocean is not substantially different than inland or freshwater. For example, the ocean has a DIC concentration of 27–29 mg/L, while the Great Lakes of North America have a DIC of 20–24 mg/L.^4–6^ Likewise, inland seas and estuaries, which have lower salinities than the ocean but higher than freshwater, also have DIC contents in that range: The Mediterranean Sea has a DIC concentration of 20–25 mg/L.^7–12^

Inspired by the natural absorptive capacity of aqueous ecosystems, some recent work has focused on exploring carbon capture from oceans.^13,14^ Most of the prominent technologies for aqueous carbon removal function via electrochemically generated pH swings, referred to as bipolar membrane electrodialysis (BPMED).^15–19^ In these systems, the CO_3_^2–^ and HCO_3_^–^ anions are converted to gaseous CO_2_ through electrochemical acidification. However, the membranes used to facilitate the pH swing can be expensive and highly susceptible to fouling—an issue caused by salt precipitation from the large ion density in natural, aqueous sources.^20–22^ Beyond electrochemical approaches, Hornbostel and co-workers investigated alternative strategies for removing dissolved CO_2_ from aqueous systems. In one study, they designed and evaluated basic microcapsules for extracting gaseous CO_2_ from simulated seawater.^23^ In another investigation, the same team examined the feasibility of using commercially available hollow fiber membranes in conjunction with caustic solvents to facilitate the diffusion and absorption of dissolved gaseous CO_2_.^24^ Both approaches were limited to capturing dissolved gaseous CO_2_ and demonstrated only minimal removal efficiency from the aqueous feedstock.

Ion exchange processes offer a promising pathway for directly removing HCO_3_^–^ or CO_3_^2–^ anions from water. In ion exchange, HCO_3_^–^ or CO_3_^2–^ anions are either adsorbed onto the surface of a solid support and exchanged for bound anions or precipitated from solution as an insoluble salt.^25–28^ Williams et al. demonstrated that Amber Jet 4400, a tertiary amine-functionalized anion exchange resin, could capture 0.07 g of HCO_3_^–^ per gram of resin.^25^ However, the use of tightly packed resin beads or gels poses unique concerns regarding the high amounts of pressure required to move water through the resin beds, making large-scale implementation energy-intensive. Hollow fibers can also be used as adsorptive membranes for the extractions of ions and do not involve the same concerns regarding pressure and scale-up.^29^ However, hollow fibers have yet to be utilized as supports for HCO_3_^–^-specific ligands, despite these advantages. These challenges highlight the need for more advanced, energy-efficient sorbent-based technologies capable of capturing CO_3_^2–^ and HCO_3_^–^ anions from aqueous sources—offering a promising complement to existing electrochemical methods.

In this work, we designed and demonstrated polymer–sorbent–grafted hollow fiber contactors as a promising approach for HCO_3_^–^ removal from aqueous sources (Figure 1a). Density functional theory (DFT) calculations were first employed to identify ligands with high affinity for HCO_3_^–^ under aqueous conditions. With the information from computational results, a guanidinium-functionalized polyguanidinium (PGA) polymer was synthesized for HCO_3_^–^ capture and grafted to commercially available polyvinylidene fluoride (PVDF) hollow fibers. PVDF hollow fiber was selected as the physical support because of its prolific use in the water treatment industry,^30^ largely due to its cost-effectiveness,^31^ compact footprint,^32^ and chemical/material robustness.^33^ A bench-scale prototype contactor module was constructed from our functionalized polymer sorbent-grafted PVDF fiber to demonstrate carbon capture from water. The system operated through a two-step process: selective ion exchange enabled adsorption of HCO_3_^–^ ions, followed by desorption, triggered by a pH swing (Figure 1b). In the first phase, adsorption occurs where HCO_3_^–^-rich water is loaded into our PGA-functionalized PVDF hollow fibers containing a sealed module. During this phase, ion-exchange occurs, swapping the counterions (e.g., halogens) bound to the guanidinium for HCO_3_^–^ anions dissolved in the aqueous feedstock. In a second phase, the guanidinium ligands are regenerated through a mild pH swing (pH ≈ 4.5), effectively releasing bound HCO_3_^–^ anions as gaseous CO_2_ and restoring the guanidinium salt. The regenerated anion in the guanidinium salt is determined by the conjugate base of the inorganic acid used in the desorption step. Our prototype demonstrated the removal of inorganic carbon from both NaHCO_3_ solutions and saltwater (e.g., sodium chloride (NaCl)). This result indicated ion selectivity of the guanidinium ligand-based polymer in the presence of NaCl, the most prominent species in seawater (∼58.5%). HCO_3_^–^ capture and release were maintained over 13 consecutive adsorption–desorption cycles, highlighting the durability of our functionalized hollow fiber sorbent contactors.

**Figure 1:**
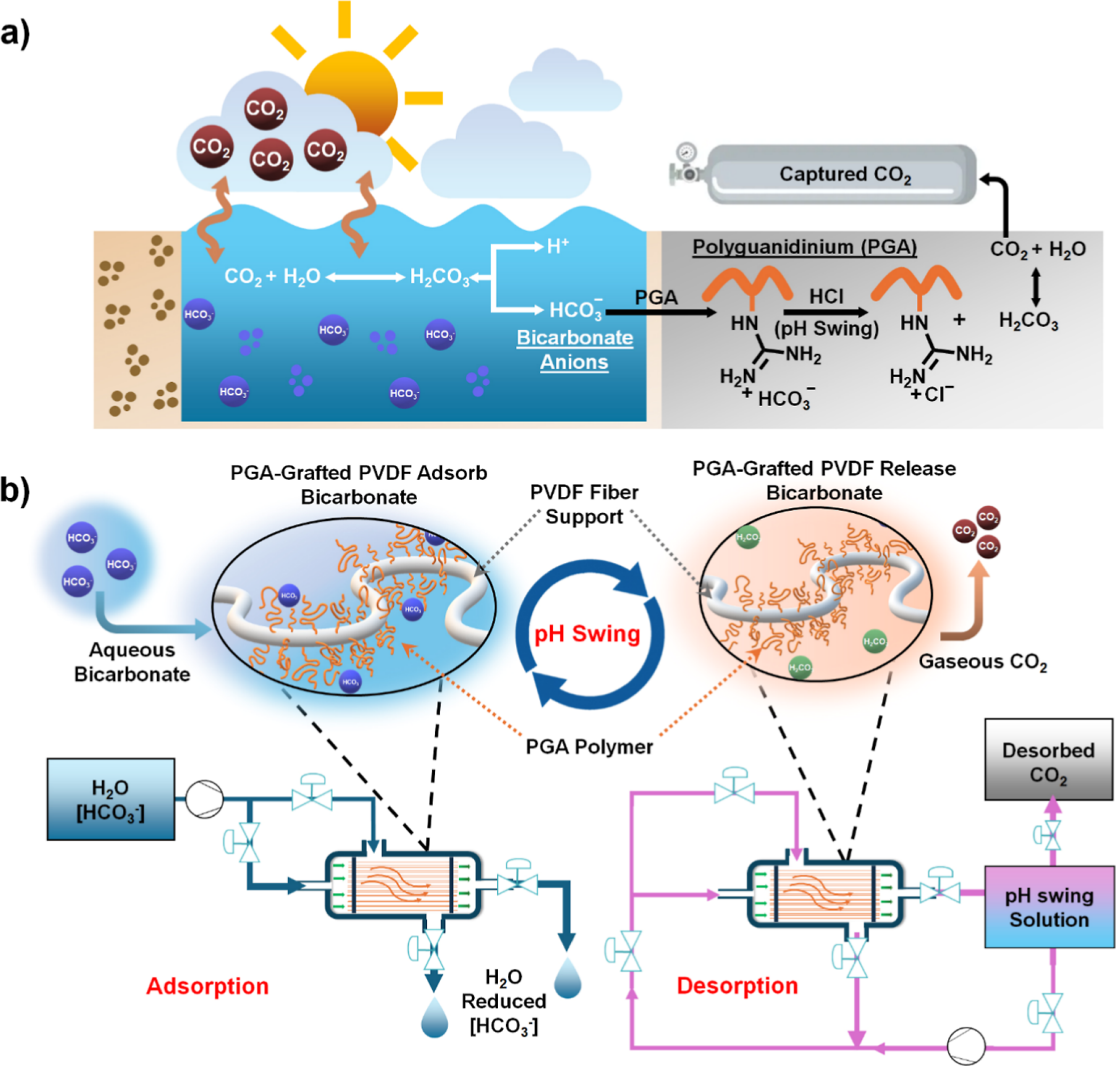
Process overview for aqueous HCO_3_^–^removal. (**a**) Diagram showing the adsorption of aqueous HCO_3_^–^ anions onto guanidinium-functionalized polyguanidinium (PGA) polymer sorbent and desorption of CO_2_ via pH swing. (**b**) PGA-grafted polyvinylidene fluoride (PVDF) hollow fiber contactors for CO_2_ separation from water via a two-step process. In the first step, the HCO_3_^–^ is adsorbed onto the guanidinium polymer selectively, such that HCO_3_^–^-reduced water elutes from the module. In the second step, a mildly acidic solution is applied to the fibers to release bound HCO3– anions as carbon dioxide (CO_2_) gas.

## Results and Discussion

### DFT Calculations for the Selection of the Ligand Structure

As the initial step of this research, we performed a qualitative computational screening to guide ligand selection. Broadly, the Hofmeister series categorizes guanidinium as a chaotropic cation, which supports the qualitative expectation of favorable interactions with anions, including oxyanions such as HCO_3_^–^.^34^ However, the series does not provide quantitative insight into the relative strength of these interactions, motivating the use of DFT calculations to compare candidate ligands under aqueous conditions.

Various nitrogen­(N)-based amine derivatives were screened via simulation to assess their HCO_3_^–^ -binding potential in aqueous environments (Figure 2a). Though ammonium-derived sorbents have been shown experimentally to bind CO_2_ and CO_2_ derivatives, we sought to identify how the chemical structure affected the binding affinity in aqueous conditions.^35–43^ DFT and semiempirical calculations were employed to evaluate the binding affinities of these candidate ligands with the HCO_3_^–^ anion under solution-phase conditions. This computational approach allowed for quantitative comparisons between the binding interactions of a diverse set of ammonium- and guanidinium-derived species with HCO_3_^–^ anions. Similar computational approaches have been widely used to analyze guanidinium–ion and oxoanion interactions, particularly for hydrogen-bonded ion–pair complexes in solution.^44,45^

**Figure 2:**
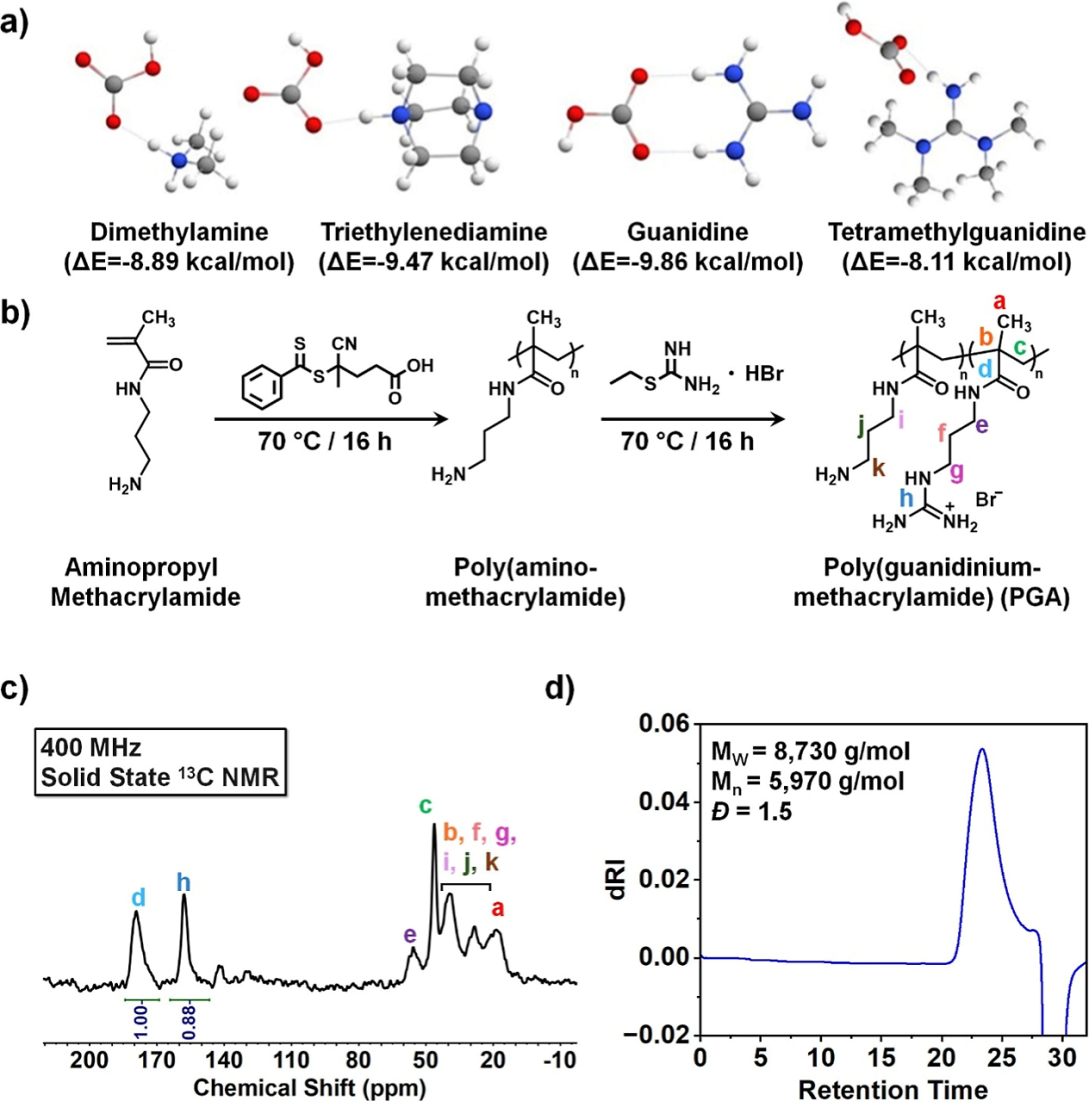
HCO_3_^–^-selective ligand selection and polymer synthesis. (**a**) DFT models calculated binding energies of several candidate ligands, modeled in their protonated states and interacting with bicarbonate as ion–pair complexes. (**b**) Reaction scheme for the polymerization and postpolymerization modification of the PGA·Br polymer. (**c**) Solid-state ^13^C NMR spectrum with relevant integrations and peaks labeled in reference to the polymer in figure (**b**). (**d**) Gel permeation chromatography (GPC) data plotted as a function of dRI against retention time. The *M*_*W*_ was identified at 8730 g/mol, *M*_*n*_ at 5970 g/mol, and *D̵*= 1.5.

To ensure algorithmic consistency, all bases were evaluated using a uniform workflow. Initial structural searches and coordination center identification were performed with the Docker algorithm. Examples of protonated amines and complexes of HCO_3_^–^ with protonated nonguanidinium and guanidinium amines are presented in Figure 2a. Notably, it was essential to employ either explicit or implicit solvent models during docking and subsequent optimizations; gas-phase calculations were found to artificially favor the migration of a hydrogen atom from the *N*-center of a base to the *O*-center of the HCO_3_^–^ ion, a process thermodynamically favorable in the gas phase but not representative for the aqueous phase.

Tables S1,S2 summarize the calculated binding energies (Δ*E*, kcal/mol) and selected topological parameters for a series of protonated amines (including methylamine) and guanidinium derivatives interacting with HCO_3_^–^. The results highlighted the superior binding performance of guanidinium-based ligands compared to conventional amines. Across the series, protonated guanidinium species consistently demonstrated the strongest binding, with Δ*E* values near −10 kcal/mol in water. This enhanced affinity derived primarily from their ability to establish two N–H···O hydrogen bonds with HCO_3_^–^, which collectively resulted in a more stabilized complex. Notably, QTAIM analysis revealed that guanidinium-based ligands exhibited lower electron density (ρ) at each individual hydrogen bond critical point relative to nonguanidinium amines, indicating that each N–H···O contact was intrinsically weaker. However, the cooperative effect of forming two hydrogen bonds in guanidinium derivatives led to overall binding energies that surpassed those of conventional amines, which engaged in only a single hydrogen bond. As a result, the total stabilization of the guanidinium framework was stronger than single bonds in nonguanidinium amines; therefore, conventional amines showed binding energies that are 1–2 kcal/mol less favorable. Progressive alkylation of the guanidine diminished the number of available N–H donors and introduced steric hindrance, ultimately reducing binding strength. For instance, fully substituted 1,1,3,3-tetramethylguanidine, possessing only a single N–H bond, demonstrated a binding affinity even lower than nonguanidinium amines. Collectively, these computational findings provided a rational foundation for the selection of saturated guanidinium ligands as the active moieties in our polymer sorbent design. They underscored that maintaining multiple cooperative hydrogen bond donors within a compact guanidinium framework was crucial for maximizing HCO_3_^–^ binding affinity, while avoiding excessive alkylation was necessary to preserve binding performance and accessibility.

### Synthesis of the Guanidine-Derived Sorbent

Building upon our computational models, nonalkylated guanidinium was selected as the ligand base for our sorbent contactors (Figure 2a). We sought to synthesize a polymer that possessed a large density of guanidinium functional groups to maximize the binding potential of the final product. The ligand-derived polymer was synthesized through a reversible addition–fragmentation chain-transfer (RAFT) polymerization reaction with *N*-(3-aminopropyl)­methacrylamide (APMA) monomer and 4-cyano-4-((phenylcarbonothioyl)­thio) pentanoic acid (CTPA) as the chain transfer agent, based on modified previously reported methods (Figure 2b).^40,46^ The resulting polyamine was converted to polyguanidinum (PGA) through postpolymerization modification with 2-ethyl-2-thiopseudourea hydrobromide to yield a polyguanidinum bromide (PGA·Br) salt. Conversion of the amine functionality to guanidinium was monitored by NMR spectroscopy. ^1^H NMR showed characteristic peaks confirming polymerization—two peaks for vinyl protons on the APMA were absent at 5.72–5.79 ppm (Figure S1). Guanidinium formation was also evidenced by the appearance of protons (a) at 3.65 ppm (Figure S1). However, the high charge density of the guanidinium-functionalized polymer slightly reduced its solubility, even in aqueous media, limiting the feasibility of solution-state ^13^C NMR, which requires relatively high concentrations to achieve adequate signal-to-noise. To characterize the percent conversion and ensure reliable quantitative integration, we examined the PGA·Br through solid-state ^13^C NMR spectroscopy.^47,48^ The resulting ^13^C NMR spectrum of PGA·Br was used to determine the ratio of amine to guanidinium functional groups inherent to the polymer (Figure 2c). To ensure quantitative integration was accurate T1 was measured using saturation recovery and the longest T1 measured to be ∼50 s. As such we used a recycle time of 300 s using a direct detection Hahn echo with ^1^H decoupling. Integration of guanidinium carbon (h) and carbonyl carbon (d) peaks at 158 and 179 ppm, respectively, in the solid-state ^13^C NMR suggested that ∼90% of the 1° amines were converted to guanidiniums. The presence of primary amine functionality (10%) was eventually necessary for grafting to the surface of the PVDF hollow fibers. After identification of the amine/guanidinium ratio, GPC was conducted to characterize the molecular weight (*M*_*w*_) and, therefore, binding capacity of the polymer (Figure 2d). Results showed an *M*_*w*_ of 8730 g/mol and an *M*_*n*_ of 5970 g/mol, suggesting that the degree of polymerization (DP), referring to the number of repeating units, was ∼24. Furthermore, end-group analysis was carried out by comparing the integral of the aromatic protons of the RAFT chain-transfer agent (δ ≈ 8.1 ppm) with that of the protons labeled “a” on the guanidinium functional group (Figure S1). This analysis yielded a DP of 33.6, corresponding to an *M*_*n*_ of 6170 g/mol. These values are consistent with the molecular weight range estimated from GPC, independently validating the polymer length and molecular weight.

### Carbon Removal Behaviors of the PGA Polymer

To evaluate the intrinsic HCO_3_^–^ binding capability of the guanidinium-functionalized PGA polymer prior to integration into the PVDF hollow-fiber contactor, we first examined HCO_3_^–^ uptake by the neat PGA·Br polymer in aqueous NaHCO_3_ solution. This experiment aimed to determine whether the polymeric guanidinium moieties could undergo ion exchange with HCO_3_^–^ and promote precipitation upon HCO_3_^–^ binding, a behavior previously observed in small-molecule guanidinium systems.^35,36^ To test this, a 50 mg/mL aqueous solution of PGA·Br and a separate 2.38 mM NaHCO_3_ solution were prepared and measured for residual bicarbonate/inorganic carbon content through a total inorganic carbon analyzer (TIC). A volume of 2 mL (100 mg) of the PGA·Br solution was added dropwise into the HCO_3_^–^ solution under ambient conditions. Within 1 min of mixing, a white precipitate began to form and was centrifuged (5–10 min) to settle from solution (Figure 3a). The formation of precipitate indicated the generation of an insoluble PGA·HCO_3_ complex via ion exchange with the original PGA·Br salt. After precipitation, the solution was again tested for changes in inorganic carbon concentration and showed a 43% removal rate, dropping from 143 ± 0.8 ppm of HCO_3_^–^ to 82.1 ± 2 ppm (Figure 3b, Table S3). When the experiment was repeated with 5 mL (250 mg) of PGA·Br solution, the removal efficiency increased to 63%, indicating that HCO_3_^–^ uptake scaled with polymer loading. Time-dependent depletion of HCO_3_^–^ concentration following polymer addition further showed that HCO_3_^–^ depletion occurred rapidly following polymer addition and reached saturation within minutes (Figure 3c, Table S3), consistent with fast ion-exchange kinetics. These results confirmed that the guanidinium-functionalized polymer effectively captures HCO_3_^–^ and supported its use in subsequent PGA–PVDF fiber contactor experiments. The bicarbonate–guanidinium interaction is expected to proceed through two possible ion-exchange pathways (Figure 3d). In the first, HCO_3_^–^ forms a 1:1 ion pair with a single guanidinium cation, replacing the original bromide counterion. In the second, HCO_3_^–^ acts as a bridging anion coordinated by two adjacent guanidinium groups, generating a dimeric complex that enhances charge stabilization and promotes precipitation. Prior work on small-molecule guanidinium systems has correlated precipitation with adsorption, reporting an uptake of approximately 1.5 mol of HCO_3_^–^ per mole of guanidinium dimer before precipitation.^35^ To assess whether a similar stoichiometry governed precipitation in the polymeric system, the mass of the PGA·HCO_3_ pellets recovered from the 5 mL (250 mg PGA) experiment was correlated with the measured decrease in bicarbonate concentration. After correcting for the molar masses of PGA·Br and HCO_3_^–^, the apparent capture ratio was calculated to be approximately 4 mol of HCO_3_^–^ per mole of precipitated PGA·HCO_3_. Although this value exceeds the reported small-molecule dimer ratio, only ∼20% of the total polymer mass added was recovered as an insoluble pellet. Given the measured dispersity of the polymer (*D̵* ≈ 1.5), we attribute the recovered precipitate primarily to lower-molecular-weight PGA fractions, which are more prone to aggregation and phase separation upon ion exchange. In contrast, higher–molecular-weight polymer chains likely remained soluble following bicarbonate binding; HCO_3_^–^ associated with these solubilized chains would neither contribute to the pellet mass nor be fully reflected in TIC measurements of the supernatant. This observation highlights a key limitation of relying on precipitation-based capture mechanisms in homogeneous polymer solutions. While ion exchange between guanidinium sites and HCO_3_^–^ clearly occurs, incomplete precipitation obscures accurate quantification of total bicarbonate uptake and complicates regeneration strategies. These findings directly motivated the subsequent immobilization of PGA onto a solid support. Tethering the polymer to PVDF hollow fibers eliminates the need for bulk precipitation, ensures continuous accessibility of guanidinium binding sites, and enables quantitative bicarbonate capture and release without loss of sorbent to the soluble phase.

**Figure 3:**
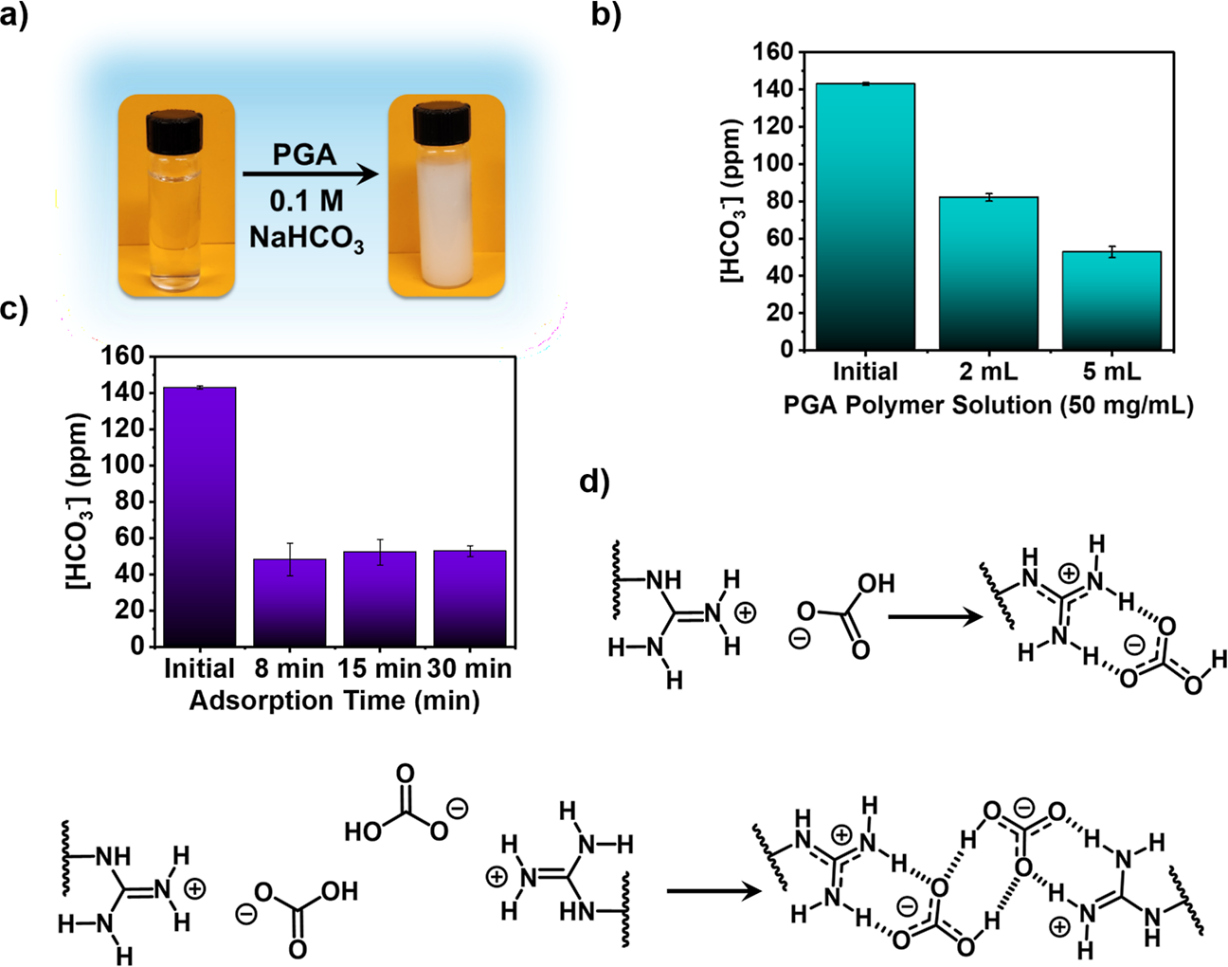
HCO_3_^–^capture using a PGA polymer. (**a**) Immediate formation of a white precipitate upon addition of PGA·Br (100 μL of a 50 mg mL^–1^ solution) into 3 mL of 0.1 M NaHCO_3_, indicating rapid ion exchange and generation of insoluble PGA·HCO_3_. (**b**) HCO_3_^–^ ion adsorption capacity of aqueous PGA polymer solution (solution concentration: 50 mg PGA dissolved in one mL of water). (**c**) Time-dependent depletion of HCO_3_^–^ concentration following polymer addition. (**d**) Proposed ion-exchange mechanisms, where HCO_3_^–^ binds either to a single guanidinium unit or as a dimer species.

### Functionalization of PVDF Hollow Fibers with PGA

Following characterization of the polymer, the next step in contactor development involved the covalent attachment of PGA·Br to solid supports.^49–53^ Typically, the chemical structure of PVDF prevents straightforward postmodification given its low solubility and lack of reactive functionalities. However, the PVDF supplier introduced reactive hydroxyl groups, which allowed for surface grafting via a straightforward, nucleophilic displacement (Figure 4a). FTIR spectroscopy of the as-received fiber showed a small, broad absorbance at 3355 cm^–1^ native to the surface hydroxyl groups (Figure 4b). To encourage grafting, conversion of the hydroxyl groups to a more electrophilic moiety was necessary to covalently link the PGA·Br polymer to the PVDF surface. For this purpose, the PVDF was first treated with a nucleophilic base (2.5 M lithium hydroxide (LiOH)) for a period of 16 h at room temperature (RT) to promote deprotonation of surface hydroxyl groups. After that time, the fibers were removed from the basic solution and were transferred to a solution of *p*-toluenesulfonyl chloride (TsCl) (0.20 g/mL) in tetrahydrofuran (THF). The fibers were allowed to stir in the solution overnight to encourage conversion of the hydroxyl groups to the more electrophilic, tosyl (Ts) species. The following day, the fibers were removed from the solution and rinsed with THF to remove any residual TsCl from the surface. The fibers were then analyzed via FTIR spectroscopy (Figure S2) and showed a decreased absorbance in the –OH region (3360 cm^–1^). Similarly, a decrease in the peak at 1728 cm^–1^ and the appearance of new peaks near the fingerprint region (691 and 575 cm^–1^) were observed. The decrease at 1728 cm^–1^ was likely due to a reduction in carbonyl (C=O) functionalities contained within the hydrophilic coating on the PVDF fibers.^54^ The new peaks, at 691 and 575 cm^–1^, were attributed to the aromatic C–H bonds on the newly added Ts functional groups. To understand how the chemical makeup of the fibers was affected during the first step of functionalization, XPS was conducted on the as-received Arkema fibers, the deprotonated fibers, and the newly tosylated PVDF fibers (Ts-PVDF) (Table S4 and Figure S3). Results from XPS showed that the as-received fibers possessed a hydrophilic coating, comprised of carbon (C), oxygen (O), and nitrogen (N). While uncoated, neat PVDF fibers should have an equal atomic ratio (1:1) of carbon to fluoride (C/F), the coated fibers from Arkema showed a 4:1 ratio of C/F, substantially higher than unmodified PVDF. After deprotonation, the F concentration increased dramatically (2.9:1) with other atomic percentages decreasing overall (Table S4). After the tosylation step, the C/F ratio was further decreased (1.8:1), indicating additional dissolution of any remaining hydrophilic coating on the fiber surface. However, the sulfur (S) concentration of the fiber increased after tosylation when compared to the deprotonated fiber. Because the Ts groups possess a sulfur atom in the chemical structure, this indicated the successful addition of Ts to the fiber surface. The final step of the fiber modification proceeded through a nucleophilic substitution mechanism where the Ts groups installed on the surface were displaced by the primary amines within the PGA·Br structure. To accomplish this, the Ts-PVDF were submerged in a solution of PGA·Br in water (15 mg/mL) and heated at 70 °C overnight. After 16 h, the fibers were removed from the viscous solution and dialyzed against deionized (DI) water to remove any of the unbound polymer from the surface of the fibers.

**Figure 4:**
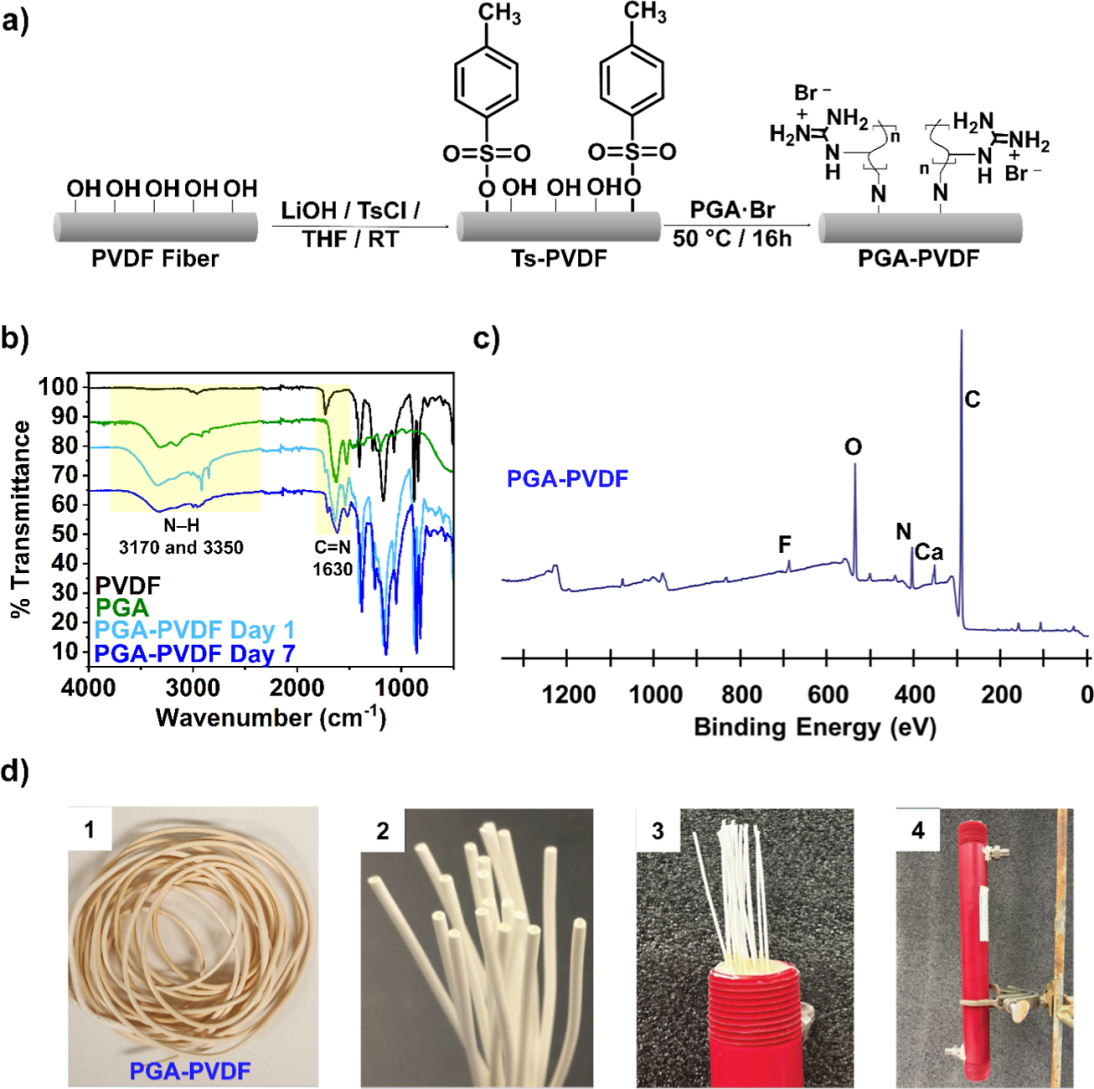
Surface functionalization of PVDF hollow fiber using PGA·Br polymer. (**a**) Schematic showing the simplified functionalization steps to generate the PGA–PVDF fiber contactors. (**b**) FTIR spectra for as-received PVDF, as-synthesized PGA, and the PGA–PVDF functionalized fibers after day 1 and day 7 of dialysis against DI water. (**c**) XPS spectrum for the PGA–PVDF functionalized contactor shows a decrease in fluorine (F) atoms and a large increase in oxygen (O) and nitrogen (N), which suggests that the addition of the PGA·Br to the PVDF surface was successful. (**d**) Images of module construction using 20 PGA–PVDF hollow fibers. (1) Coils of PGA–PVDF fibers from the scaling process, which generated 20 functionalized fibers (14″ length). (2) Loose hollow fiber contactors trimmed and aligned. (3) PGA–PVDF contactors were drawn through a custom-made module with cured epoxy to create a seal. (4) Fully assembled custom-made module containing PGA–PVDF hollow fiber contactors.

### Characterization of PGA–PVDF Hollow Fiber Contactors

The PGA-functionalized PVDF (PGA–PVDF) fibers were analyzed via FTIR by monitoring changes in absorbance over the dialysis period (Figure 4b). The emergence of N–H and C=N stretches were observed at 3170/3350 cm^–1^ and 1630 cm^–1^, respectively, and were attributed to the PGA. For comparison, the FTIR spectra of as-received Arkema PVDF and neat PGA·Br polymer are included in Figure 4b. An initial decrease in the normalized absorbance of the fiber peak at 1630 cm^–1^ (C=N) between day 1 and day 2 of dialysis (Figure S4) was observed, likely due to the removal of loosely bound polymer. However, after 7 days of dialysis, the spectra remained stable, indicating retention of the covalently grafted PGA·Br. XPS of the fiber was conducted after day 7 of dialysis and showed an increase in the N % and decrease in the F % (Figure 4c and Table S4). Likewise, the C % and S % values were altered after PGA·Br addition. These changes were correlated with the addition of the amine-rich, PGA·Br polymer, which increased C, O, and N percentages. The decrease in S % indicated the displacement and removal of Ts groups from the fiber surface during the nucleophilic substitution reaction (Table S4).

To visualize the effects of PGA·Br functionalization on the surface morphology of PVDF, SEM was conducted on both the as-received fiber from Arkema and the PGA-functionalized fiber. An image of the as-received fiber (Figure S5) showed a relatively smooth surface with minimal deviations. Likewise, the images acquired of the PGA-functionalized fibers showed no substantial morphological deformations and an overall smooth surface (Figure S5). Macroscopic images of the fibers (Figure 4d) showed a slight change in color but no major deformities, which could affect the potential for HCO_3_^–^ binding.

After characterization of the PGA–PVDF, we sought to identify the overall binding capacity of the resultant fiber. Since the carbon-capture capabilities were directly related to the number of ligands on the surface of the fiber, an understanding of the functional density was necessary. TGA was completed as a method to determine how much the mass of the PVDF fiber was affected by the addition of PGA·Br as well as to characterize the degradation temperature (Figure S6). A TGA experiment was conducted by heating the PGA–PVDF fibers to 100 °C, a temperature previously demonstrated to promote the release of guanidinium-sorbed CO_2_,^55^ and holding at that temperature for 45 min to stabilize the weight and remove the influence of residual water on the overall mass. The mass of the fiber was recorded at the 30 min mark, as indicated in the representative TGA plot (Figure S7). This heat-and-hold protocol was applied to fibers at each modification stage. Notably, there was a large decrease in mass when comparing the Ts-PVDF fibers with the as-received, hydrophilic fibers. This information aligned well with the results from XPS, which suggested removal of a soluble coating during the deprotonation and tosylation steps of fiber functionalization. In the final step where PGA·Br was covalently bound to the surface, there was a mass increase of 0.007 mg (7 μg) corresponding to the addition of the polymer ligand (Figure S7). The Ts-PVDF and the final PGA–PVDF intermediates were chosen to describe the functional density as an attempt to limit the influence of further coating dissolution on the difference in mass. Using this mass gain, the functional density was calculated as a mass ratio of PGA/PVDF at ∼2 mg/g (0.2%). Further calculations were conducted to identify the number of ligands per unit of mass by converting the moles of polymer to moles of ligand using the DP. This conversion yielded a value of 4.6 × 10^18^ repeat units per gram of PVDF and 4.1 × 10^18^ guanidiniums per gram of PGA–PVDF (90% guanidinium). Extrapolation of the ligand functionality was conducted to identify the theoretical binding capacity of the PVDF fiber contactors. The maximum binding capacity was calculated to 4.1 × 10^18^ HCO_3_^–^ anions per gram of PVDF contactor accounting for a 1:1 binding of guanidinium to HCO_3_^–^. To construct the benchtop module, the functionalized PGA–PVDF contactors were drawn through a 12-in. length PVDF pipe (Figure 4d). With the new module fabricated, the total mass of the 20 fibers in the module was calculated to ∼2.7 g by scaling the measured dimensions of the functionalized fiber used in the TGA experiments to 10.5 in.. Further extrapolation yielded a theoretical maximum binding capacity of 8.2 × 10^18^ HCO_3_^–^ anions per module (20 fibers at 10.5″ active length).

### Carbon Removal Behaviors of the PGA–PVDF Fiber Contactor Module

The ability of PGA–PVDF fibers to chemisorb CO_2_, in the form of HCO_3_^–^, was evaluated in small-scale gas-phase experiments using dry and humid CO_2_ in a modified TGA system. The PGA–PVDF sorbs CO_2_ based on a chemisorption process, which, unlike CO_2_ physisorbents with neutral sorption sites, only exhibit CO_2_ sorption properties in water/moisture-rich conditions vital for the conversion of CO_2_ gas to HCO_3_^–^ ions at the gas/solid interface. Approximately 1 cm of functionalized fiber was placed in a platinum TGA pan and monitored for mass changes during exposure to controlled CO_2_ environments. Before adsorption measurements, each fiber underwent an activation step in which it was heated to 100 °C under N_2_ to remove adventitious moisture or inorganic carbon accumulated during storage (Figure S8). In contrast, when the experiment was repeated using humidified CO_2_, the adsorption behavior changed markedly (Figure 5a). The same activation phase was performed to equilibrate the mass of the fiber before exposure to the humid CO_2_ source. Unlike in the dry CO_2_ phase, the weight increased rapidly; we speculate that this is due to adsorption of both HCO_3_^–^ and H_2_O onto the fiber surface between 300 and 600 min, as shown in Figure 5a. To calculate the amount of HCO_3_^–^ adsorption, the change in weight was corrected to account for H_2_O (Figure S9) and equated to an uptake of 0.18 mmol CO_2_/g PGA–PVDF, substantially increased from the dry CO_2_ experiment. These results confirm that the PGA–PVDF fibers preferentially bind HCO_3_^–^ over neutral CO_2_, as expected from the guanidinium ion-exchange mechanism.

**Figure 5:**
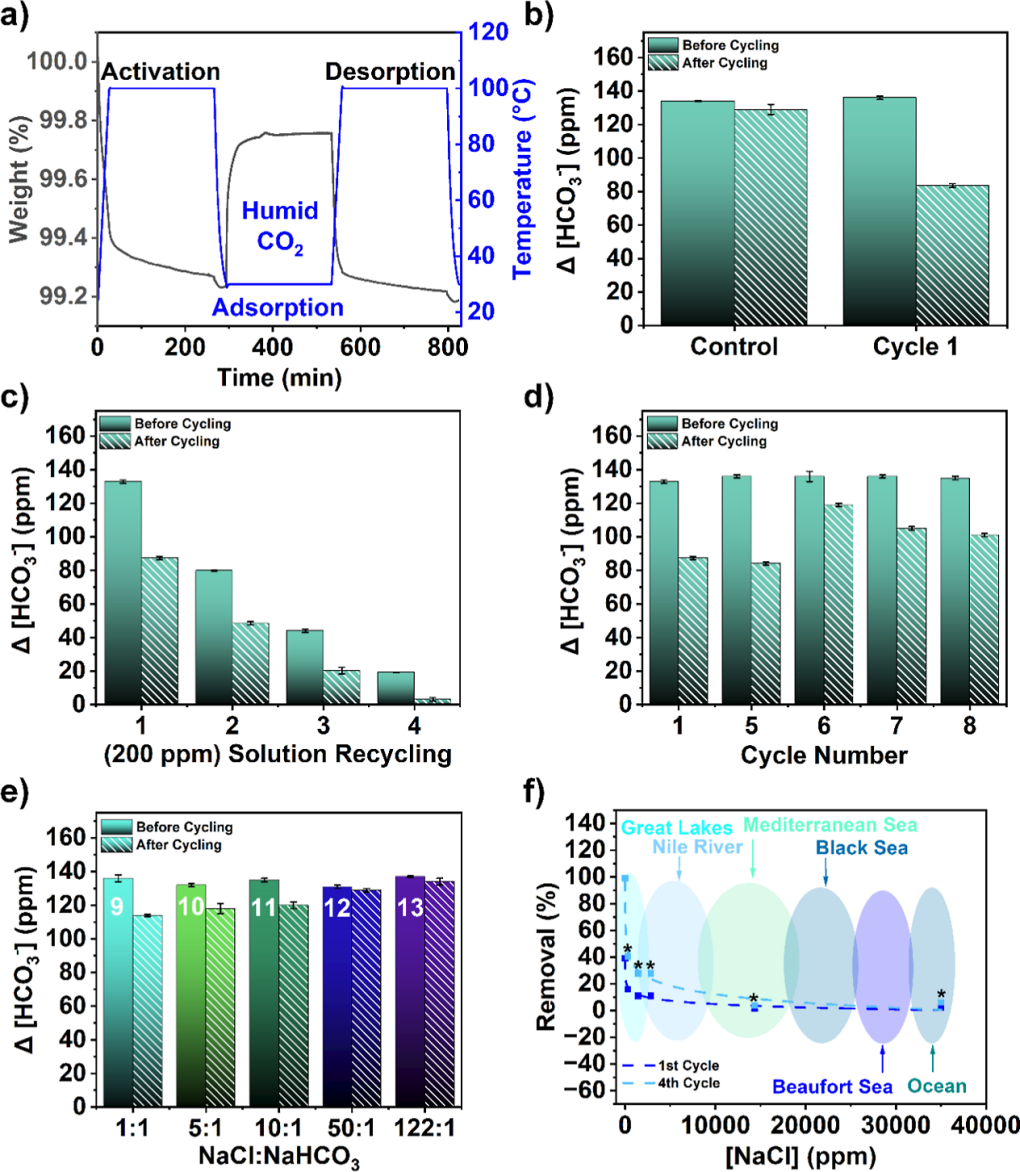
Bench-top model performance and inorganic carbon removal of PGA–PVDF hollow fiber contactors. (**a**) CO_2_ uptake experiment under humid conditions (50%RH, 30 °C) for the PGA–PVDF via the TGA measurement. (**b**) Changes in HCO_3_^–^ concentration for PGA–PVDF and as-received fibers (control) performed with NaHCO_3_ in deionized (DI) water. (**c**) Changes in HCO_3_^–^ concentration during a recycling experiment where solution 1 was fed back through the PGA–PVDF contactors 3 additional times (total 4 cycles). After cycle 4, 98% of the inorganic carbon had been removed from the HCO_3_^–^ solution. (**d**) Changes in HCO_3_^–^ concentration for 4 additional adsorption cycles performed with NaHCO_3_ in deionized (DI) water as a measure of stability. (**e**) Changes in HCO_3_^–^ concentration for adsorption performed with 1:1, 5:1, and 10:1 mixtures of NaCl/NaHCO_3_ by mass. Inset numbers show the cycle number correlated with Table S8. (**f**) HCO_3_^–^ removal percentages for mixtures of NaCl/NaHCO_3_ ranging from 0:1 to 122:1 with corresponding natural bodies of water plotted for comparison. Removal percentages for the fourth cycle were linearly extrapolated based on data from the solution with an NaCl/NaHCO_3_ concentration of 0:1. This linear relationship was assumed to remain constant with respect to NaCl/NaHCO_3_ concentration. Projected values are marked with asterisks.

To investigate the binding capacity of our PGA–PVDF fiber contactor module, different compositions of aqueous solutions, with and without the presence of NaCl, were assessed. All solution compositions used in binding and competitive ion studies are listed in Table S5. Adsorption experiments were conducted under closed-batch conditions for 24 h to prevent atmospheric CO_2_ intrusion and to ensure the system reached near-complete adsorption equilibrium, which is particularly important given that the smaller-sized hollow-fiber-containing module contained only 100 mL of solution in volume. Although hollow fiber contactors are typically operated under continuous flow, the batch configuration enabled controlled, quantifiable measurements of HCO_3_^–^ uptake in this initial study; future work will focus on evaluating the system under steady-state flow and at larger scales.

To assess bicarbonate binding in the absence of competing ions, the fibers were first exposed to 100 mL of a 200 ppm NaHCO_3_ solution prepared in Milli-Q deionized water. After 24 h of gentle agitation, the conductivity of this solution was measured to monitor adsorption, and it was observed that the solution’s conductivity increased by 26.0 ± 0.4 μS. Although removal of anions from solution would generally be expected to decrease conductivity, the observed increase aligns with an ion-exchange process: as HCO_3_^–^ binds to the guanidinium groups on the polymer, it displaces smaller and more mobile halide counterions, which then enter solution and raise the overall conductivity (Table S6). This trend was maintained throughout the remainder of the adsorption experiments (2–13) where the conductivity increased after each adsorption cycle. In contrast, solutions exposed to control (ungrafted) PVDF fibers exhibited a slight decrease in conductivity (−15.5 ± 0.5 μS), likely due to physical adsorption of the HCO_3_^–^ onto/into the as-received PVDF and the absence of any counterion exchange. Though conductivity measurements provided preliminary evidence of ion exchange, quantitative verification of HCO_3_^–^ removal was obtained through TIC analysis of the same solutions. The same solutions tested for conductivity were subsequently analyzed for their TIC values. The initial 200 ppm of NaHCO_3_ solution exposed to the PGA–PVDF functionalized contactors showed a decrease in inorganic carbon content, changing from 133 ± 1 ppm to 87.3 ± 1 ppm over the period of testing (24 h), marking a 34% reduction in overall inorganic carbon (Figure 5b). In contrast, the control PVDF fiber with no guanidinium functionality showed no significant change in TIC values after a 24 h exposure period, changing from 134 ± 0.4 ppm to 129 ± 3 ppm, confirming that HCO_3_^–^ uptake arises from guanidinium functionality rather than from nonspecific adsorption.

To evaluate the regeneration capability of the PGA–PVDF sorbent via a pH-swing process, bicarbonate-saturated fibers were introduced to a slightly acidic solution of HCl (pH 4.5) in a flow process (Figure S10). As desorption proceeded, the effluent pH rose toward neutrality, reflecting release of HCO_3_^–^ back into solution through the ion exchange process. During this ion-exchange step, bound HCO_3_^–^ is displaced by Cl^–^, and the liberated HCO_3_^–^ is immediately protonated to form H_2_CO_3_, which subsequently decomposes to CO_2_ and water. The pH was intermittently adjusted back to 4.5 when the solution approached a pH of 7 to maintain desorption conditions. The pH versus time plots for all regeneration experiments performed in the 13 cycles are shown in Figure S11. Regeneration typically required 70–100 min and consumed ∼16 μL of 12.1 M HCl per cycle, equivalent to ∼350 mol of HCl per mol of HCO_3_^–^ released. Notably, this process converts PGA·HCO_3_^–^ to PGA·Cl rather than restoring the original PGA·Br; bromide originates from the polymer synthesis and is exchanged only during the first adsorption cycle. Subsequent cycles involve reversible exchange between Cl^–^ and HCO_3_^–^, with the identity of the regenerated anion governed by the inorganic acid used.

To assess overall removal capability, the adsorption–desorption cycle was repeated until the inorganic carbon concentration approached zero. It was observed that 4 cycles were required to lower the carbon content from 133 to 3 ppm, a 98% reduction (Figure 5c **and** Table S7). This result suggests that increasing the number or length of fibers—or increasing sorbent mass—could reduce the number of cycles required to achieve comparable removal. Sorbent stability was evaluated in cycles 5–8 using fresh 200 ppm of NaHCO_3_ solutions (Figure 5d, Table S8). Across these cycles, HCO_3_^–^ removal ranged from 12% to 39% (Table S8). The variability likely reflects gradual fiber degradation or incomplete regeneration with repeated cycling. Nonetheless, the fibers consistently demonstrated reversible HCO_3_^–^ capture and release over eight cycles, confirming the feasibility of the PGA–PVDF contactor design for repeated operation.

After establishing HCO_3_^–^ capture performance in pure NaHCO_3_ solutions, adsorption cycles 9–13 evaluated the selectivity of PGA–PVDF fibers in increasingly saline conditions. NaCl was introduced at mass ratios of 1:1 up to 10:1 relative to NaHCO_3_ (Figure 5e). At a 1:1 NaCl/NaHCO_3_ ratio, the inorganic carbon decreased by 22.0 ± 2 ppm (16% removal), lower than the 34.0 ± 2 ppm (25% removal) observed in the salt-free cycle 8. As NaCl concentration increased, HCO_3_^–^ removal efficiency declined due to competitive occupation of guanidinium binding sites by Cl^–^ (Table S9). However, even at the highest salinity tested (10:1 NaCl/NaHCO_3_), the PGA–PVDF fiber contactor achieved 11% HCO_3_^–^ removal, indicating that passive inorganic carbon capture in brackish water remains feasible. To place these results in an environmental context, we compared the contactor performance with salinity levels representative of natural water bodies (Figure 5f). Extrapolation of multicycle operation suggests that >25% of dissolved inorganic carbon could be removed after four adsorption–desorption cycles even under seawater-like salinity. These findings highlight the robustness of guanidinium-based ion exchange under competitive ionic conditions and underscore the potential of PGA–PVDF contactors for carbon removal across a wide range of aqueous environments—from freshwater to brackish and marine systems.

## Conclusions

This work establishes guanidinium-functionalized PVDF hollow fiber contactors as a viable and regenerable platform for selective HCO_3_^–^ capture from aqueous environments. Guided by the DFT-based ligand design, we synthesized a PGA sorbent, covalently grafted it to PVDF supports, and demonstrated that the resulting fibers bind HCO_3_^–^ through a robust ion-exchange mechanism. Gas-phase experiments confirmed the PGA sorbent’s preferential affinity for HCO_3_^–^ over neutral CO_2_. The bench-scale prototype achieves an initial HCO_3_^–^ removal of 34%, which increases to 98% after four adsorption–desorption cycles. The functionalized fibers maintained aqueous stability over at least 13 cycles in model NaHCO_3_ solutions, and regeneration via a mild pH swing enabled complete sorbent recovery with minimal acid input, converting bound HCO_3_^–^ to CO_2_ and restoring the active guanidinium sites. Importantly, the system sustains selective performance in the presence of competitive chloride ions; carbon removal remained above 10% even at high (10:1) NaCl/NaHCO_3_ ratios, underscoring its relevance for natural bodies of water. While this work focuses on guanidinium as the ligand of choice, further investigation into alternative amine- and ammonium-based ligands may reveal additional binding motifs and enhance HCO_3_^–^ affinity. Likewise, although PVDF served as the support material for this module, opportunities exist to leverage other fibers and micro/nanostructured materials for surface modifications to increase functional group density and improve ion-exchange capacity. The role of competitive ion binding (e.g., sulfates) remains unexplored yet important to the development of sorbents for use in aqueous carbon capture. This may also be relevant to future wastewater research given the known binding behavior of phosphate ions to guanidinium.^56,57^ In conclusion, this study demonstrated the feasibility of using surface-modified contactors for carbon removal from aqueous sources, laying the groundwork for future advancements in selective ion exchange as a viable approach to NETs.

## Supplementary Material



## Data Availability

All data needed to evaluate the conclusions in the paper are present in the paper and/or the Supporting Information.

## Notes

Materials, methods, equipment, chemical analysis, bicarbonate capture analysis, theoretical methods, DFT calculations, polymer synthesis, PVDF fiber functionalization, gas sorption test, conductivity measurements, experimental setup for testing the ion exchange capacity of PGA polymers, NMR spectra, FTIR spectra, XPS, SEM images, TGA analysis, pH measurements, sorbent density characterization on PVDF surfaces, regeneration experiments, and quantum-chemical calculation (PDF)
