# Supplementary material for: Aqueous Carbon Capture Using Guanidinium-Functionalized Hollow Fiber Sorbent Contactors
Source: JACS Au. 2026 Mar 30;6(4):2303–14. doi: 10.1021/jacsau.5c01639 (PMC13126169; doi:10.1021/jacsau.5c01639)
Supplement: Supplementary file 1 [file au5c01639_si_001.pdf]

Supporting Information

Aqueous Carbon Capture Using Guanidinium-Functionalized Hollow Fiber Sorbent Contactors

Mary K. Danielson,[1,2#] Nicholas Gregorich,[1#] Mary H. Irwin,[1,3] Cyril Pepple,[4] Anton S. Pozdeev,[1,5] Alexander S. Ivanov,[1,6] Joshua Damron,[1] Zachary Coin,[1] Tarryn Trick,[7] Tomonori Saito,[1,3,8] Ramesh Bhave,[1] João Marreiros,[4] Ryan P Lively,[4] Syed Z. Islam,[1*] Md Anisur Rahman[1*]

[1]Chemical Sciences Division, Oak Ridge National Laboratory, Oak Ridge, TN 37831, USA

[2]Circular Bioeconomy Systems Convergent Research Initiative, University of Tennessee Oak Ridge Innovation Institute, Knoxville, TN 37996, USA

[3]Materials Science and Engineering, University of Tennessee, Knoxville, TN 37996, USA

[4]School of Chemical and Biomolecular Engineering, Georgia Institute of Technology, Atlanta, GA 30332, USA

[5]Department of Chemical & Biomolecular Engineering, Vanderbilt University, Nashville, TN, 37235, USA

[6]Department of Nuclear Engineering, University of Tennessee, Knoxville, TN 37996, USA

[7]Department of Chemistry, Washington University in St. Louis, St. Louis, MO 63130, USA

[8]Bredesen Center for Interdisciplinary Research and Graduate Education, University of Tennessee, Knoxville, TN 37996, USA

Equal co-author/contributor

*Corresponding Author E-mail: islamsz@ornl.gov; rahmana1@ornl.gov

This PDF file includes:

Supplementary Text

Tables S1-S9

Figures S1-S11

Supplementary Text

Materials and Equipment. *N*-(3-aminopropyl)methacrylamide and ethyl carbamidothioate hydrobromide were purchased from Ambeed and were used without further purification. 4,4'-Azobis(4-cyanovaleric acid) (V-501) initiator, lithium hydroxide (LiOH), *p*-toluene sulfonyl chloride (TsCl), and 4-cyano-4-((phenylcarbonothioyl)thio)pentanoic acid (CTPA) were purchased from Sigma-Aldrich chemicals and were used without further purification. Dimethyl formamide (DMF), tetrahydrofuran (THF), methanol (MeOH), and acetate buffer were purchased from Thermo Fisher. ASTM D-1141-98 (13) Formula A (Lot: TL-ASTM-24061) Sea Salt was acquired from Lake Products Company LLC. Carbon dioxide standard (0.1 M $NaHCO_3$) Orion 950206 was acquired from Thermo Scientific. Sodium chloride (NaCl) anhydrous, free-flowing, Redi-Dri, ReagentPlus 99% (CAS: 7647-14-5) was acquired from Sigma-Aldrich. Hydrochloric acid (HCl) A114C-212 Certified ACS Plus (CAS: 7647-01-0, Lot 140105) was acquired from Fisher Scientific. Polyvinylidene fluoride (PVDF) loose hollow fiber membranes were acquired from Arkema, Inc. (600 µm ID, 1,200 µm OD). Schedule 80 PVDF (0.957" ID, 1-5/16" OD) piping was acquired from McMaster-Carr. LOCTITE EA 9462 structural adhesive was procured from Henkel. Masterflex Digital Gear Pump (Model No. 75211-70, 36-3600 rpm, 0.1 HP) and MICROPUMP GA Series high performance suction-shoe pump head (model: GA-T23.PFS.A) were acquired from Avantor. LSE Vortex Mixer REF 6775 was acquired from Corning.

Chemical Analysis. ^{1}H NMR spectra were recorded with a Bruker instrument (400 MHz) and signals were measured in deuterium oxide (D_2O) using a residual proton signal value of 4.79 ppm from the solvent. ^{1}H NMR spectroscopy was successfully conducted at 10 mg mL^{-1}, a concentration at which the polymer remained fully soluble. Solid state ^{13}C NMR spectra were also recorded on a Bruker instrument at 400 MHz. Gel permeation chromatography (GPC) was conducted on an analytical GPC from Agilent utilizing aqueous columns with light scattering and refractive index detectors from Wyatt technology. Calibration was completed using polyethylene oxide standards (EasyCal) from Agilent technologies and were run in sequence with the PGA sample. The molecular weights of the standards ranged from 435 g/mol to 32,200 g/mol. GPC measurements were performed at a concentration of 2.5 mg mL^{-1}, well within the confirmed solubility limit of the polymer, ensuring reliable elution behavior. Fourier transform infrared spectroscopy (FTIR) was collected using an attenuated total reflectance (ATR) sampling method

with a diamond crystal on a Perkin Elmer Spectrum Two instrument. XPS data was collected from a Thermo Scientific Nexsa G2 instrument on a section near the middle of each tested fiber. The fiber was attached to the XPS sample holder using double-sided carbon-tape. For each sample, a single analysis point was defined on each fiber and a wide energy range survey spectrum was acquired to determine all elements present. Next, a set of narrow energy range core level spectra were acquired for each element identified. SEM images were collected from a Tescan Mira 3 at 10 kV, 1,000, 20,000, and 50,000 x magnification. Measurements from thermogravimetric analysis (TGA) were conducted using the heat-and-hold protocol (isothermal method) on a TGA Q50 from TA Instruments. This method aided in determination of the functional density of the modified fibers where the mass of the fiber was collected after a period of drying at 100 °C to remove any influence from ambient humidity.

Bicarbonate Capture Analysis. Total inorganic carbon (TIC) analysis was done with a CM5330 Acidification Module and CM5017 CO_2 Coulometer. Samples of 10 mL were loaded into the cell at 25 °C and met with 10 mL of 2M HCl. Ambient air was used for the carrier gas flowing at 100 mL/min. Conductivity was measured using an Oakton CON 150 waterproof portable meter and probe. Prior to measurement, the probe was calibrated for expected μS/cm ranges. pH was measured using a VWR pHenomenal pH 1100 L meter. Prior to performing a regeneration experiment, the pH electrode was calibrated with buffer solutions representing pH 4, 7, and 10. During regeneration, the meter was set to log pH measurements every 1 min for 80 to 100 minutes.

Theoretical Methods:

We applied a sequential computational approach consisting of a global minimum (GM) search, high-level geometry optimization with harmonic frequency analysis, molecular docking, and chemical bonding analysis using quantum-theory of atom in molecules (QTAIM) (**Table S1, and Figure 2a**).[1]

Initially, the GM search for the protonated N-based species was conducted using the CREST program (version 3.0.2)[2] with the GFN2-xTB semi-empirical method.[3] This step aimed to comprehensively explore conformational space and identify the lowest-energy conformer.

In the next stage, the geometries obtained from the GM search were re-optimized, and harmonic frequencies were calculated using DFT as implemented in the ORCA software (version 6.0.1).[4]

We applied the PBE0 hybrid functional[5] with the D3BJ dispersion correction,[6, 7] combined with the def2-TZVPD basis set.[8, 9] and SMD solvation model with water as a solvent.[10] Here, this theoretical level is referred to as PBE0-D3BJ/def2-TZVPD. Similar optimization and frequency analyses were performed for HCO_3^- at the PBE0-D3BJ/def2-TZVPD level.

Following these separate optimizations, molecular docking simulations between the optimized amine and bicarbonate structures were performed using ORCA. Docking calculations were carried out at the GFN2-xTB level with the ALPB implicit solvent model (water as solvent),[11] ensuring both host (amine) and guest (bicarbonate) species remained flexible during the docking process.

In the final computational step, the docked complex was reoptimized, and harmonic frequencies were recalculated at the PBE0-D3BJ/def2-TZVPD level of theory. To provide deeper insight into the nature of the ligand–bicarbonate interactions, chemical bonding analyses were conducted using QTAIM as implemented in Multiwfn 3.8.[12] To validate the chosen DFT protocol for ion-pair geometries and hydrogen-bond metrics in aqueous phase, we benchmarked our approach against the experimentally determined crystal structure of guanidinium bicarbonate.[13] The PBE0-D3BJ/def2-TZVPD optimized structure reproduces key geometric descriptors of the experimentally observed guanidinium–bicarbonate binding motif, including the average N···O hydrogen-bond distance and the characteristic O–C–O and N–C–N bond angles (Table S2). This agreement supports the reliability of the employed computational setup for describing guanidinium–bicarbonate interactions.

Polymer (PGA) Synthesis. *N*-(3-aminopropyl)methacrylamide (10 g, 70 mmol, 1 eq) was combined with 4-cyano-4-((phenylcarbonothioyl)thio)pentanoic acid (0.06 g, 0.2 mmol, 0.003 eq) and V-501(4,4′-Azobis(4-cyanovaleric acid)) initiator (0.01 g, 0.04 mmol, 0.00055 eq) and stirred at 70 °C in a 1:1 solution of DMF:acetate buffer (pH 5.5) overnight (16 h). After that time, the polymer was transferred to a dialysis bag with a MW cutoff of 500 Da and dialyzed against water for 4 days to remove unreacted monomer and small chain oligomers. To quaternize the polymer and add guanidine functional groups, ethyl carbamidothioate hydrobromide (40 g, 200 mmol, 3 eq) was added to the polymer in H_2O. The reaction was stirred at 40 °C in a 5:1 mixture of DMF:H_2O overnight. The resulting polymer was again dialyzed against H_2O (500 Da cutoff) to purify and contained a mixture of primary amines and guanidine functional groups.

PGA-PVDF Functionalization. The PVDF hollow fibers were acquired from Arkema company and were known to contain a concentration of surface-level hydroxyl functionalities. The PVDF was activated through conversion of the surface-level hydroxyl groups to tosyl-groups (**Figure 4a**). To complete this alteration, the PVDF fibers were first treated with a 2.5M aqueous solution of lithium hydroxide (LiOH) by stirring overnight at room temperature. This process conferred additional –OH groups while deprotonating existing –OH groups. The fibers were then transferred to a solution of *p*-toluene sulfonyl chloride (TsCl) in THF (150 mg/mL) and stirred overnight. After that time, the fibers were rinsed thoroughly in MeOH to remove excess TsCl before transferring to a solution of 15 mg/mL of the PGA in H_2O. Again, the fiber was stirred overnight to complete the addition of the polymer. The fibers were removed from the polymer solution the following day and washed in pure H_2O for 4 days, changing the solution periodically, to remove unbound polymer from the fiber surface.

Gas Sorption Tests:

Dry CO_2 Adsorption Experiment. The TGA experiment was carried out using a TA Instruments TGA550. Prior to testing, the fibers were immersed in an aqueous storage solution to avoid potential degradation reactions of the functional nano coating. The samples were dried to the touch on a dry paper wipe prior to TGA analysis. The 'dried' fiber samples were placed in a 100 μL platinum sample pan and pre-saturated with dry N_2 at 30 °C before adsorption. The TGA furnace temperature was initially ramped at 3 °C/min to 100 °C to thermally activate the fibers. The fibers were then cooled down under N_2 flow until reaching a temperature of 30 °C. Once cooled down to 30 °C the adsorption step was initiated, CO_2 molecules diffused into the porous structure of the fibers and interacted with active sites, resulting in a mass gain assumed to be solely due to CO_2 uptake. Following the adsorption step was a desorption step where the system was purged with dry N_2 at 100 °C, 100 mL/min for 120 min and any physically adsorbed CO_2 was removed. This was followed by a 5 °C/min ramp to 30 °C and equilibration at 30 °C to prepare the sample for subsequent analysis. The PVDF-Guanidine fibers showed small amounts of CO_2 uptake during the adsorption phase. These results highlighted the importance of conducting humid CO_2 adsorption studies to gain deeper insight into the interactions between CO_2 and guanidine functional groups. This procedure was adapted from a method reported recently in the literature.[14]

Humid CO_2 Adsorption Experiment. TGA was carried out using a TA Instruments TGA550. The pre-dried fiber samples were placed in a 100 μL platinum sample pan and pre-saturated with humid N_2 (dew pt. ~ 18.9 °C) at 30 °C before humid CO_2 was introduced for the adsorption step. A dew point generator, LI-COR LI-610, was used to control the humidity of the inlet gas. The humidified gas was then fed directly into the furnace, where the sample was placed. The TGA furnace temperature was initially ramped at 3 °C/min to 100 °C to thermally activate the fibers. The fiber was then pre-saturated with water vapor where the sample was exposed to 100 mL/min humid N_2 (50% RH) for 240 min. This pre-saturation step helped to simulate realistic atmospheric conditions in which CO_2 is rarely present without water vapor. Moreover, this step was utilized to decouple the mass uptake of water from CO_2 in the experiment. Following the pre-saturation step, the feed gas was switched to humid CO_2 (50% relative humidity (RH)) at 100 mL/min for 240 min at 30 °C. In this step, HCO_3^- ions were formed at the solid gas interface when CO_2 and water molecules were adsorbed onto the fibers in proximity to the PGA active sites.

Dry = N_2 + 400ppm CO_2, 0% RH, T=30 ºC

Humid = N_2 + 400ppm CO_2, 50% RH, T=30 ºC

The mass gained in the adsorption step is assumed to be CO_2. To study the reversibility of the adsorption process, the system was purged with dry N_2 at 100 °C, 100 mL/min for 240 min. This desorption step removed any physically adsorbed CO_2 and any loosely bound water molecules. This was followed by a 5 °C/min ramp to 30 °C and equilibration at 30 °C to prepare the sample for subsequent analysis. This was followed by a 5 °C/min ramp to 30 °C and equilibration at 30 °C to prepare the sample for subsequent analysis. This procedure was adapted from a method reported recently in the literature.[14]

The CO_2 and water uptakes at the sample level can be calculated based on the integration of the LI-COR signals which afford an accurate measure of the water and CO_2 concentrations present in the gas feed downstream from our sample. The CO_2 adsorption capacity was determined by integrating the breakthrough curve of the CO_2 signal against that of a square advancing front of 400 ppm CO_2, a reasonable determination given the small dead volume of the TGA analysis system.

Bicarbonate Adsorption Tests. Adsorption of bicarbonate was performed in different compositions with and without the presence of NaCl. The compositions of all solutions used in this study are shown in **Table S3**. For standalone PGA polymer adsorptions were performed with 24 mL of 2.38 mM NaHCO3 loaded into a 30 mL scintillation vial. Varying volumes (2, 5 mL) of PGA polymer were injected into the solution and stirred for an amount of time (8, 15, 30 min). The PGA polymer concentration is 50 mg·mL^{-1}. After stirring, the solutions were centrifuged to separate polymer from solution. Solutions were evaluated for changes in HCO_3^- concentration via TIC analysis. The results of these experiments are shown in **Table S2**.

For module-based adsorptions, 100 mL of solution was loaded into the hollow fiber-containing module and capped to prevent solvent leakage. The loaded module was then mounted to the vortex mixer and gently agitated for 24 hours. After 24 hours, the solution was dispensed from the module and evaluated for changes in pH and TIC.

Regeneration Experiment. The regeneration of the hollow fiber membranes was performed for each cycle of adsorption in this study. For the regeneration process, a 500 mL solution of HCl in deionized water was prepared with a pH of 4.5. The solution was pumped through the shell and lumen side of the hollow fibers at 100 mL/min for approximately 70 to 100 minutes for each regeneration experiment. The schematic of this two-pump setup is shown in **Figure S10**. The pH changes during each desorption cycle are shown in **Figure S11**.

Table S1: Binding energies of various amine-derivative ligands. Calculated binding energies (ΔE, kcal/mol; PBE0-D3/def2-TZVPD level) and topological (QTAIM) parameters for the interaction between protonated N-bases and HCO_3^-.

**Protonated N-bases**	**Binding energy ΔE, kcal/mol**	**QTAIM parameters**		
		**ρ**	**Δρ**	**H(r)**
**Methylamine**	-8.73	0.0610	0.1287	-0.0162
**Dimethylamine**	-8.89	0.0598	0.1292	-0.0153
**Triethylamine**	-9.18	0.0598	0.1288	-0.0152
**Triethylenediamine**	-9.47	0.0609	0.1296	-0.0159
**Piperidine**	-8.36	0.0554	0.1246	-0.0128
**Pyridine**	-9.81	0.0716	0.1282	-0.0233
**Guanidine**	-9.86	0.0392	0.1148	-0.0037
		0.0392	0.1146	-0.0037
**1-methylguanidine**	-10.27	0.0399	0.1155	-0.0030
		0.0377	0.1124	-0.0040
**1,1-Dimethylguanidine**	-10.29	0.0392	0.1147	-0.0037
		0.0374	0.1120	-0.0037
**1,1,3-Trimethylguanidine**	-10.02	0.0239	0.0897	0.0022
		0.0391	0.1156	-0.0036
**1,1,3,3-Tetramethylguanidine**	-8.11	0.0392	0.1136	-0.0038

Table S2. Comparison of experimental (single-crystal X-ray) and computed (PBE0-D3BJ/def2-TZVPD) structural parameters for guanidinium bicarbonate (structure shown above for atom labeling).

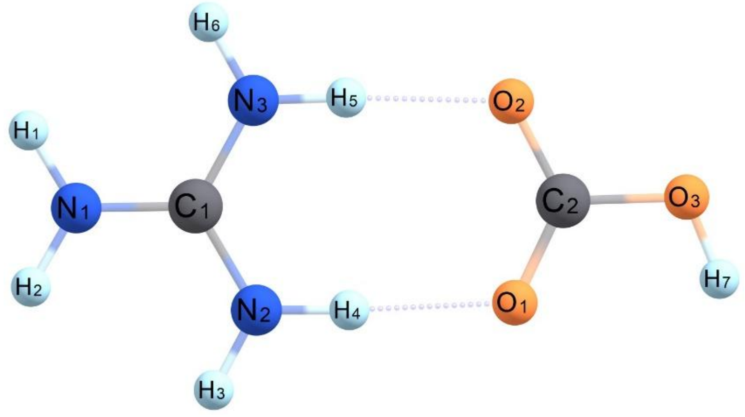

	Experimental crystallographic parameters (single-crystal XRD)	Theoretical parameters
**Bond lengths, (Å)**		
Average N-H…H	2.87(5)	2.84
O(1)-C(2)	1.238(2)	1.23
N(1)-C(1)	1.325(2)	1.33
**Bond angles, (°)**		
O(1)-C(2)-O(2)	124.9(2)	126.9
N(1)-C(1)-N(3)	119.4(2)	119.9
N(1)-C(1)-N(2)	120.8(2)	119.8

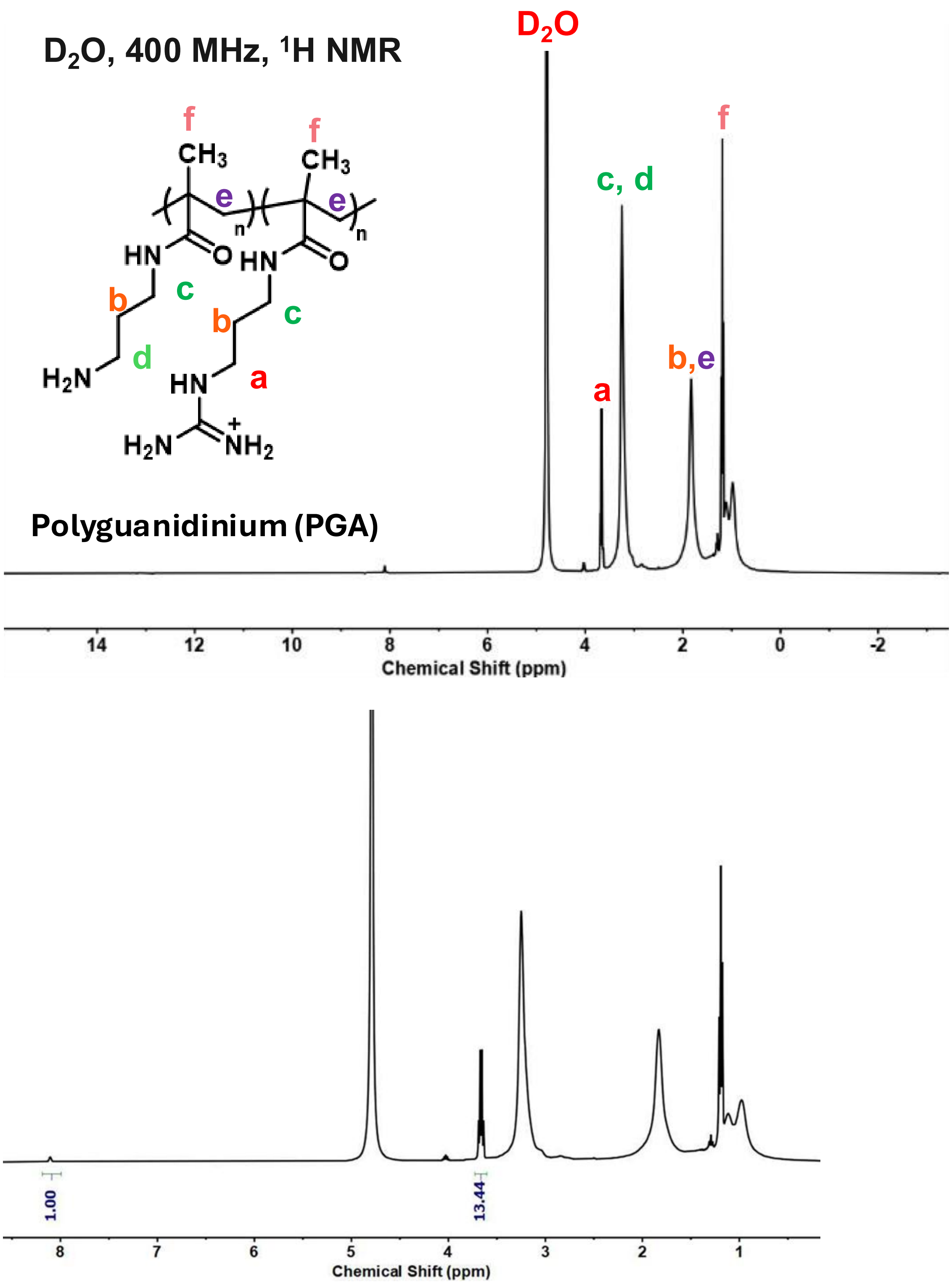

Figure S1: ^{1}H NMR spectrum of the PGA polymer in D_2O (10 mg/mL) with relevant peaks labelled. ^{1}H NMR showed complete conversion from acrylamide monomer to polymer, observed through disappearance of vinyl proton peaks. Further characterization was needed to distinguish

between amine and guanidinium functionalities. End group analysis was conducted to calculate the molecular weight of the polymer using the integration of aromatic protons from chain transfer agent (4-cyano-4-((phenylcarbonothioyl)thio)pentanoic acid) at 8.1 ppm and the protons labelled "a" on the guanidinium functional group (integrations are shown in the bottom ^{1}H NMR spectrum). The degree of polymerization (DP) was calculated to be 33.6 with an M_n of 6,170 g/mol which aligns well with the results from GPC experiments.

Table S3: Changes in TIC for starting and ending solutions of cycles using freshly prepared, 200 ppm $NaHCO_3$ solution.

Solution Composition	Volume of Polymer Sorbent (mL)	Time (min)	HCO_3^- Initial (ppm)	HCO_3^- Final (ppm)	ΔHCO_3^- (ppm)	% Removal	$\frac{mg\ HCO_3^-}{mg\ sorbent}$
200 ppm $NaHCO_3$	2	30	143 ± 0.8	82.1 ± 2	-60.9 ± 2	43	0.61
	5	30		52.8 ± 3	-90.2 ± 3	63	0.36
	5	15		52.2 ± 7	-90.6 ± 7	63	0.36
	5	8		48.1 ± 9	-96.7 ± 9	68	0.38

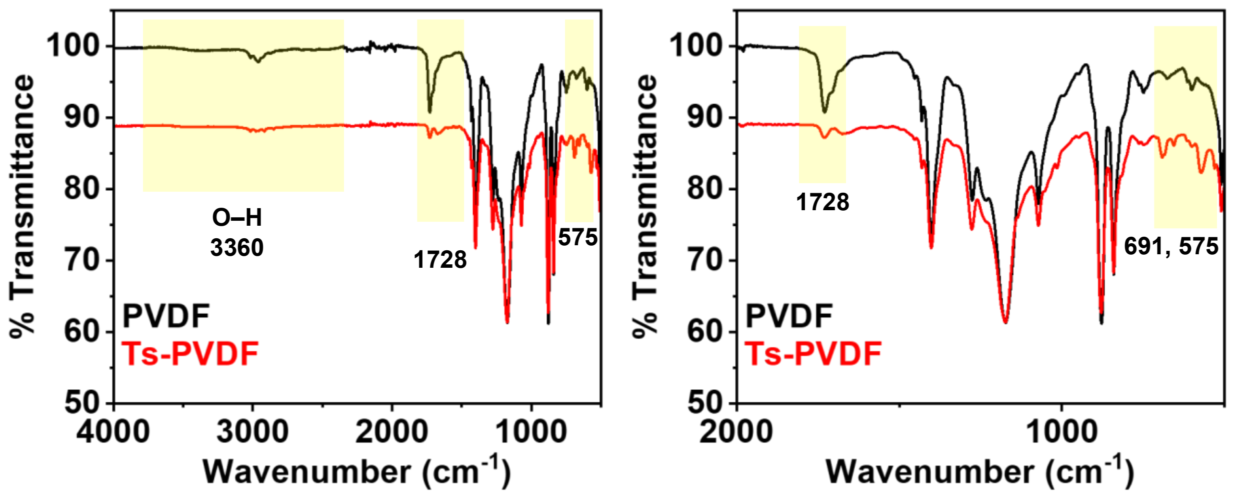

Figure S2: FTIR spectra for the PVDF and Ts-PVDF fiber contactors. Peak absorbance in the –OH region (3360 cm^{-1}) and the absorbance at 1728 cm^{-1} decreased between the PVDF and Ts-PVDF, likely corresponding to removal of a hydrophilic coating/sizing installed by Arkema Corporation. The appearance of new peaks near the fingerprint region (691 and 575 cm^{-1}) were attributed to the aromatic C-H groups inherent to the chemical structure of Ts.

Table S4: Atomic percentages identified by XPS for each step in the PVDF functionalization process. Carbon (C), oxygen (O), fluoride (F), nitrogen (N), sulfur (S), and chloride (Cl) are reported here as atomic percentages. A noticeable increase in F% was observed during the first 3 steps (PVDF->PVDF-Deprotonated->Ts-PVDF) corresponding to removal of the Arkema coating. Most notably is the increase in S% between PVDF-Deprotonated and Ts-PVDF, indicative of successful conversion of the surface hydroxyl groups, and the increase in N% between the Ts-PVDF and PGA-PVDF which suggested successful tethering of the PGA polymer to the PVDF surface.

Fiber	C	O	F	N	Si	S	Cl
**Hydrophilic PVDF**	60.9	14.2	14.5	3.3	2.9	2.7	0.7
**PVDF-Deprotonated**	63.6	5.9	22.3	5.5	0.5	0.1	0.1
**Ts-PVDF**	57.2	7.3	32.8	1	0.3	1.1	0.2
**PGA-PVDF**	70.3	12.5	3.9	8.9	2.1	0.4	0.2

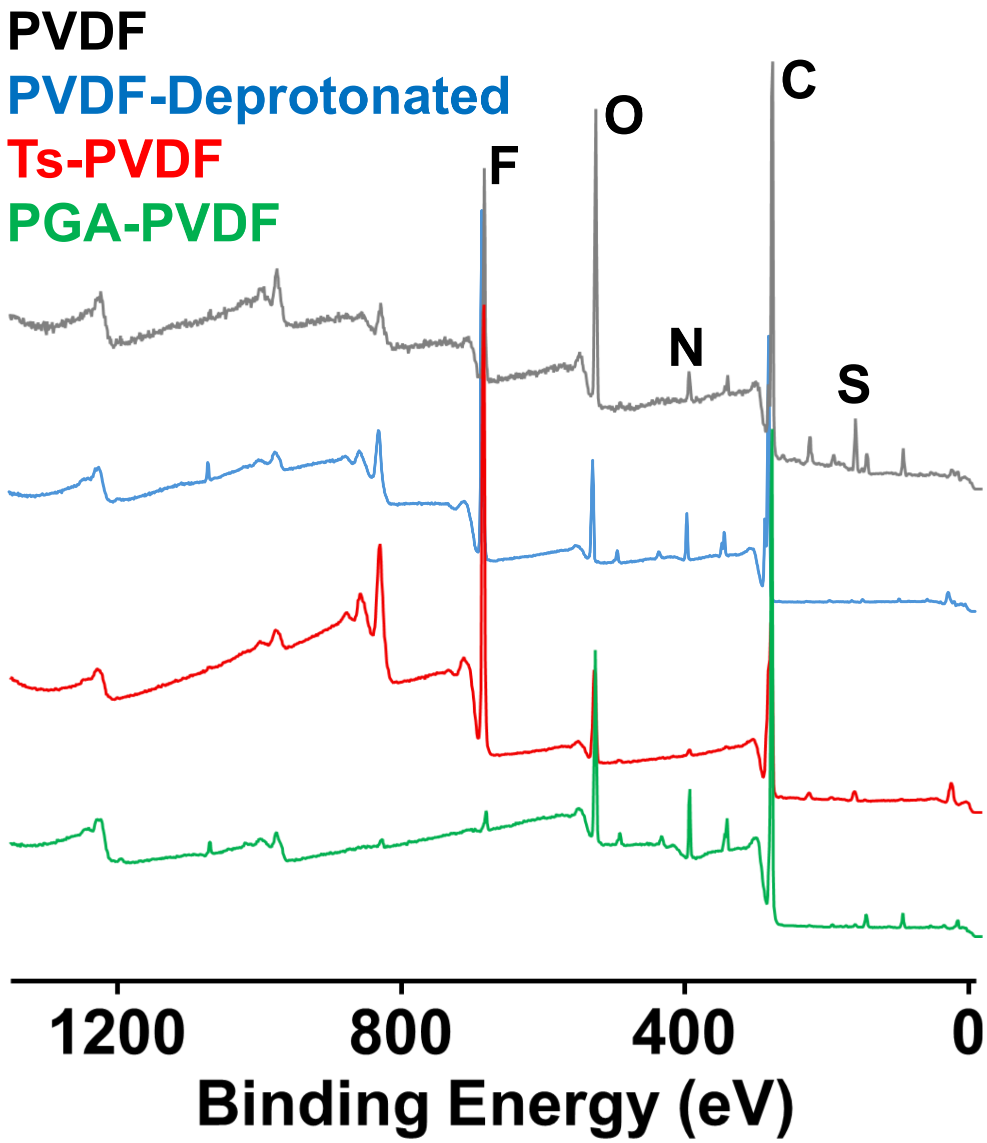

Figure S3: XPS spectra for each phase of the PVDF surface functionalization process. Spectra shown above are consistent with the values calculated in Table S2 where S% increased between PVDF-Deprotonated and Ts-PVDF and N% increased between Ts-PVDF and PGA-PVDF.

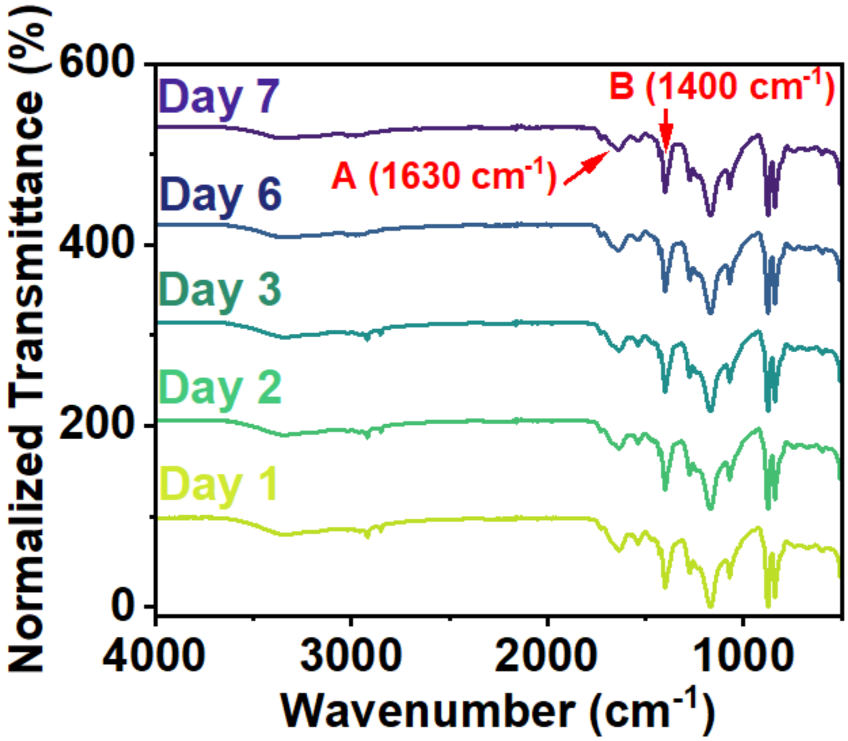

Figure S4: FTIR of the PGA-PVDF fiber contactors over 7 days of dialysis in DI water. Peaks labelled A and B were used to calculate relative changes in absorbance over the testing period and verify whether polymer shedding was occurring. A was identified as the guanidinium peak and B was utilized as benchmark given its presence on the as-received fiber. On Day 1 the A:B ratio was 2.99 and decreased to 2.95 on Day 2. After Day 2, this ratio remained stable over the course of the next 5 days. Polymer shedding was considered to have completed after Day 2 of dialysis.

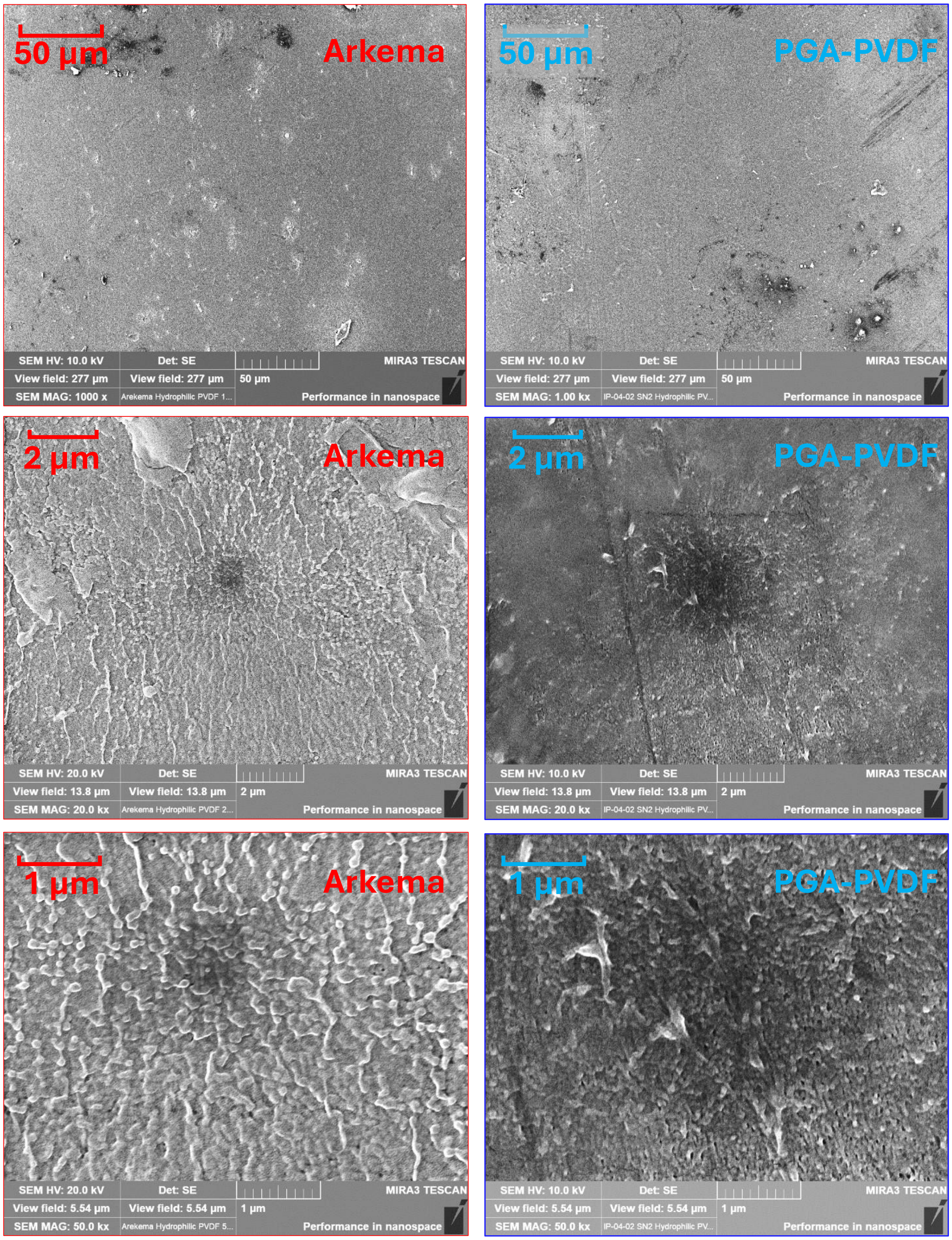

Figure S5: SEM images of Arkema PVDF and functionalized PGA-PVDF. SEM images of the as-received PVDF shows a largely uniform surface (left column). SEM images of the functionalized PGA-PVDF surface also shows uniformity and no evidence of surface damage caused by the functionalization process.

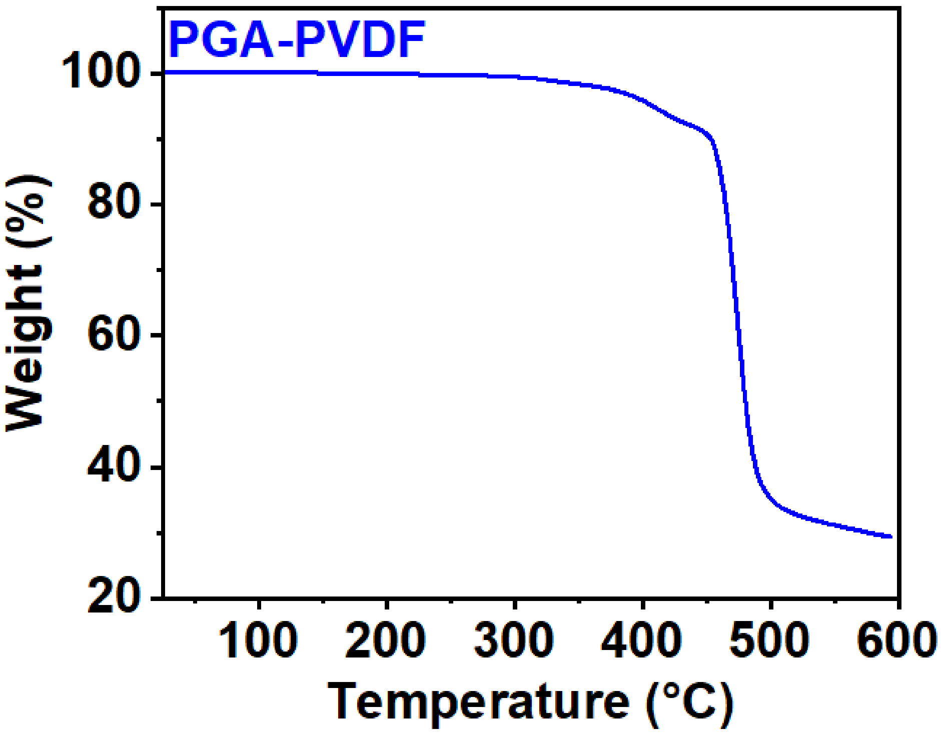

Figure S6: Thermogravimetric analysis (TGA) of PGA-PVDF. TGA analysis showed that degradation of the polymer occurred at over 300 °C, well within operating temperatures of our desired application.

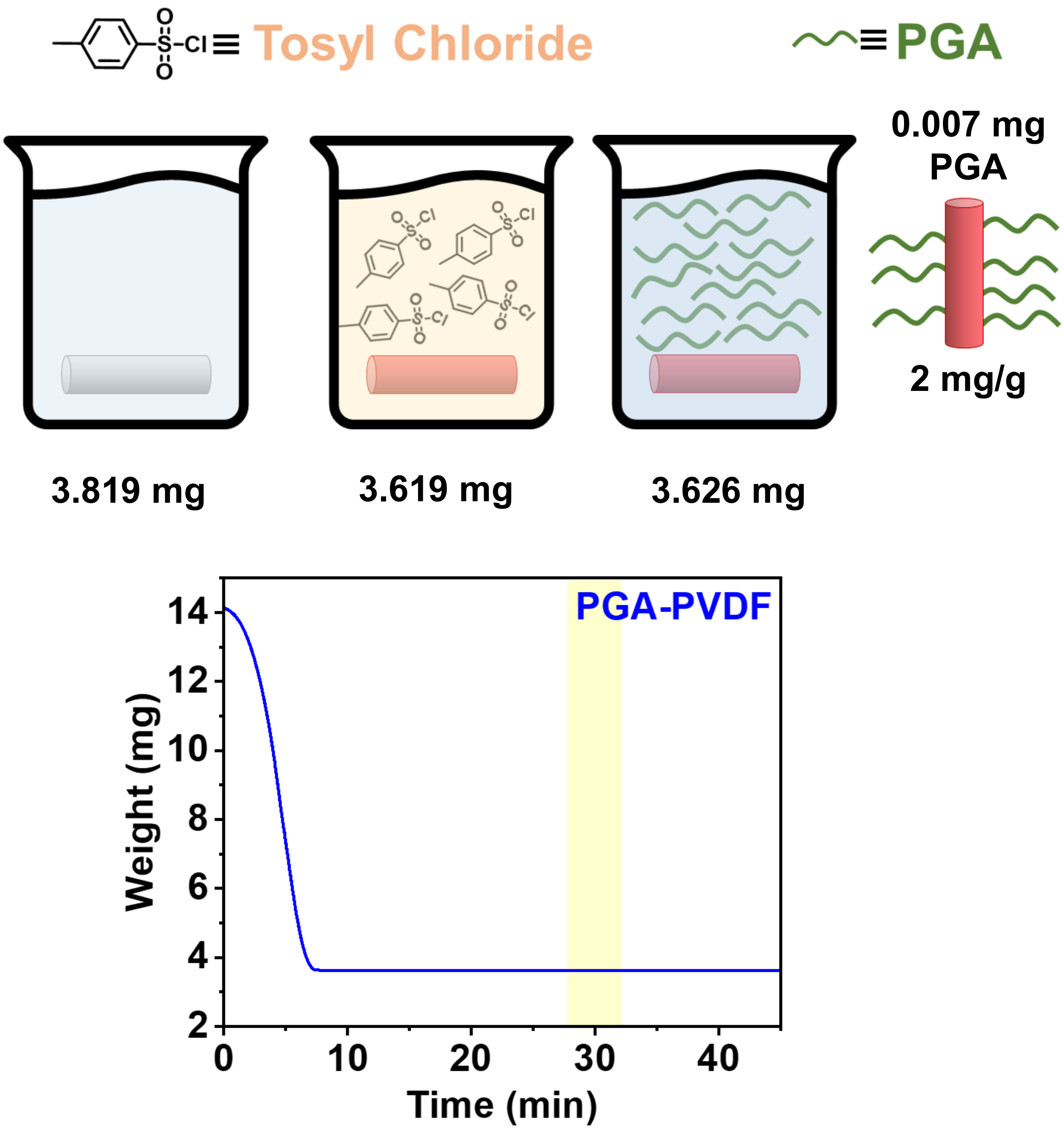

Figure S7: Schematic demonstrating the experiment utilized to characterize functional surface density of the PGA-PVDF where the mass was collected at each step during the functionalization process. The inset TGA curve below shows that the mass was collected after 30 minutes of drying to ensure reproducibility of the results. Final functionality was calculated through comparison of the PGA-PVDF to the Ts-PVDF intermediate.

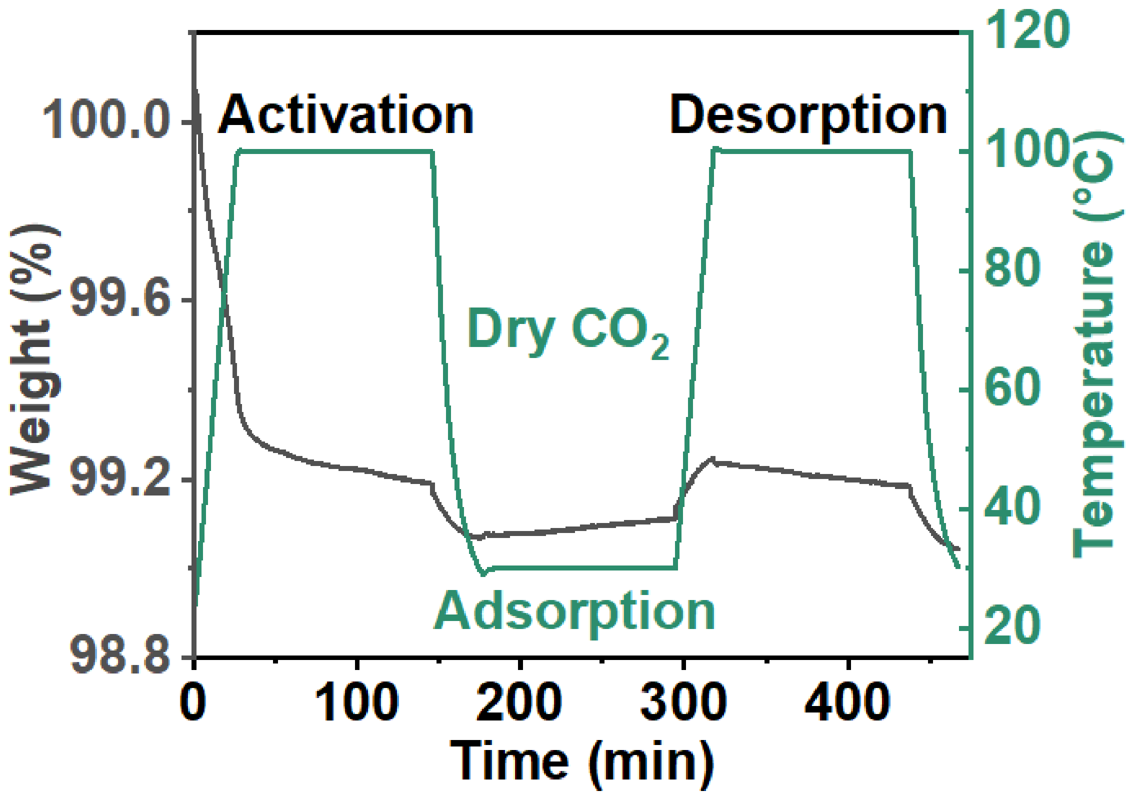

Figure S8: Dry CO_2, TGA adsorption experiment of PGA-PVDF. Dry CO_2 adsorption experimental data from TGA showed a small uptake of CO_2 between 200-300 minutes, equivalent to 0.01 mmol of CO_2/g of PGA-PVDF. This small uptake was expected to occur in dry conditions where very little of the inorganic carbon exists as bicarbonate.

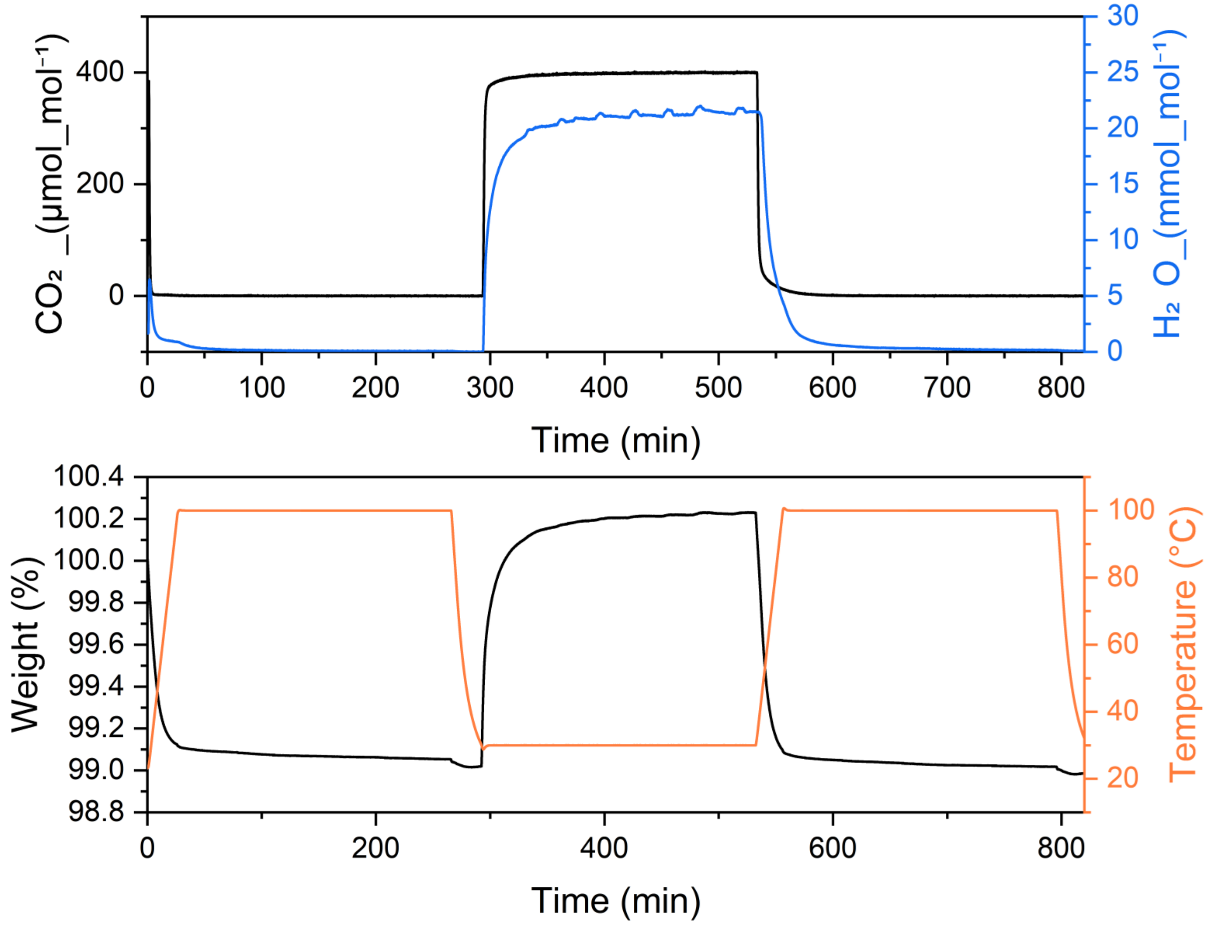

Figure S9: Humid CO_2, TGA adsorption experiment of PGA-PVDF. These spectra display the raw, collected data corresponding to Figure 5a in the main text. This data describes the adsorptive behaviors of the PGA-PVDF before correction for water adsorption by the fiber.

Table S5. Solution compositions containing $NaHCO_3$ and NaCl used in this study.

**Water Composition (wt.%)**	**NaCl (ppm)**	**NaCl (mmol)**	**$NaHCO_3$ (ppm)**	**CO_2 (ppm)**	**CO_2 (mmol)**
**200 ppm $NaHCO_3$**	0	0	200	104	2.38
**1:1 NaCl:$NaHCO_3$**	200	3.42	200	104	2.38
**5:1 NaCl:$NaHCO_3$**	1000	17.1	200	104	2.38
**10:1 NaCl:$NaHCO_3$**	2000	34.2	200	104	2.38
**50:1 NaCl:$NaHCO_3$**	10000	171.1	200	104	2.38
**122:1 NaCl:$NaHCO_3$**	24530	419.7	200	104	2.38

Table S6: Changes in conductivity for all 13 cycles analyzed in this study.

Fibers	Cycle	Solution Composition	Conductivity (time 0 h) (µS/cm)	Conductivity (time 24 h) (µS/cm)	ΔConductivity (µS/cm)
Control PVDF		200 ppm $NaHCO_3$	189 ± 0.2	174 ± 0.5	-15 ± 0.5
Guanidinium Functionalized PVDF	1	200 ppm $NaHCO_3$	194 ± 0.3	220 ± 0.2	+26.0 ± 0.4
	2	200 ppm $NaHCO_3$ *Recycled*	121 ± 0.2	129 ± 0.7	+8.0 ± 0.7
	3		68.4 ± 0.03	77.0 ± 0.3	+8.6 ± 0.3
	4		29.7 ± 0.02	36.9 ± 0.1	+7.2 ± 0.1
	5	200 ppm $NaHCO_3$	192 ± 0.1	234 ± 1	+42 ± 0.7
	6		191 ± 0.2	196 ± 0.2	+5 ± 0.3
	7		190 ± 0.3	195 ± 0.5	+5 ± 0.5
	8		190 ± 1	196 ± 0.3	+6 ± 1
	9	1:1 $NaCl:NaHCO_3$	530 ± 1	560 ± 1	+30 ± 1
	10	5:1 $NaCl:NaHCO_3$	1780 ± 2	1680 ± 2	-100 ± 3
	11	10:1 $NaCl:NaHCO_3$	3420 ± 30	3300 ± 0	-120 ± 30
	12	50:1 $NaCl:NaHCO_3$	15,100 ± 40	14,300 ± 30	-800 ± 50
	13	122:1 $NaCl:NaHCO_3$	34,800 ± 60	31,900 ± 0	-2900 ± 60

.

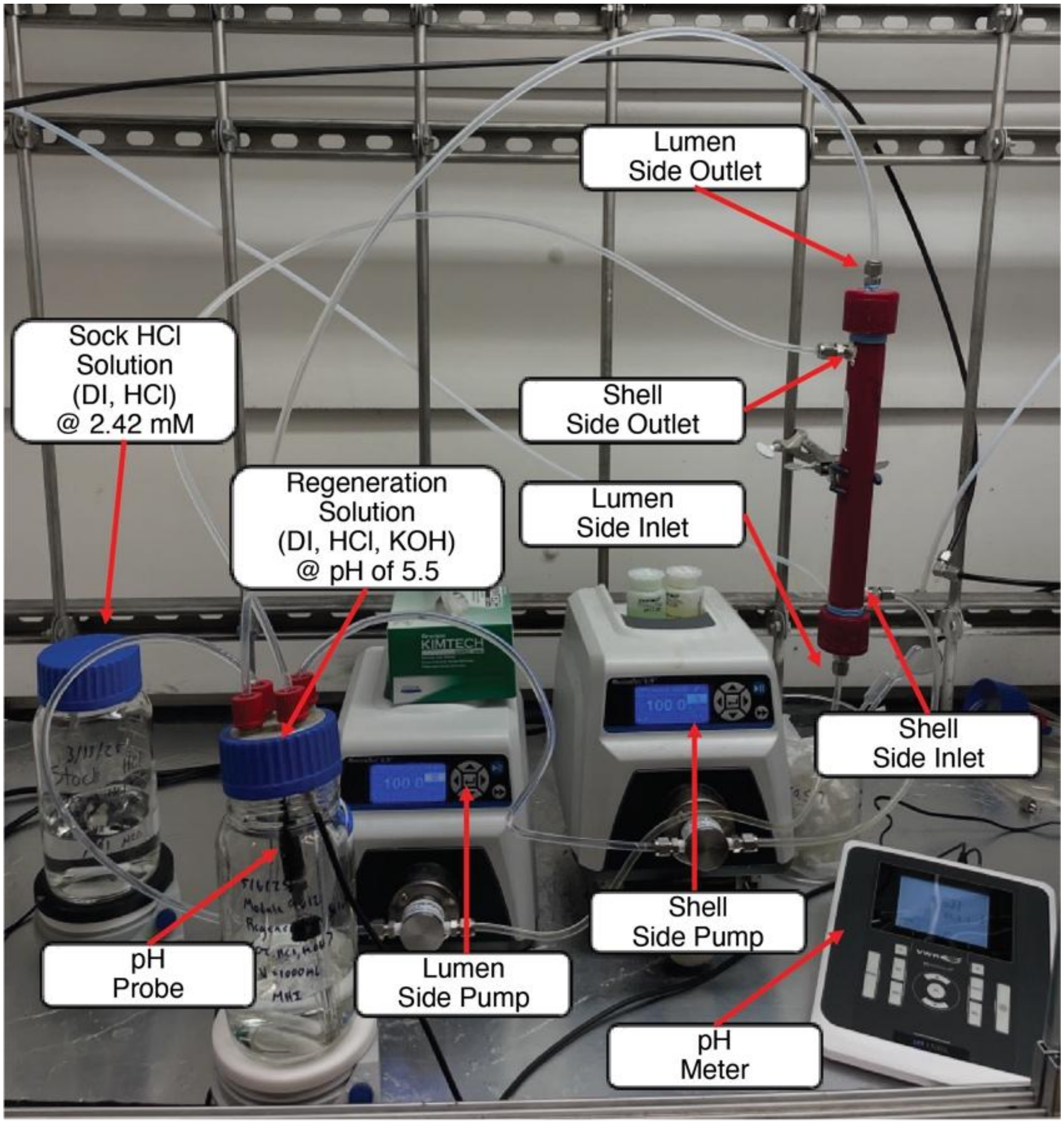

Figure S10: Configuration for two-pump system pumping HCl solution (pH = 4.5-5.5) for the regeneration process.

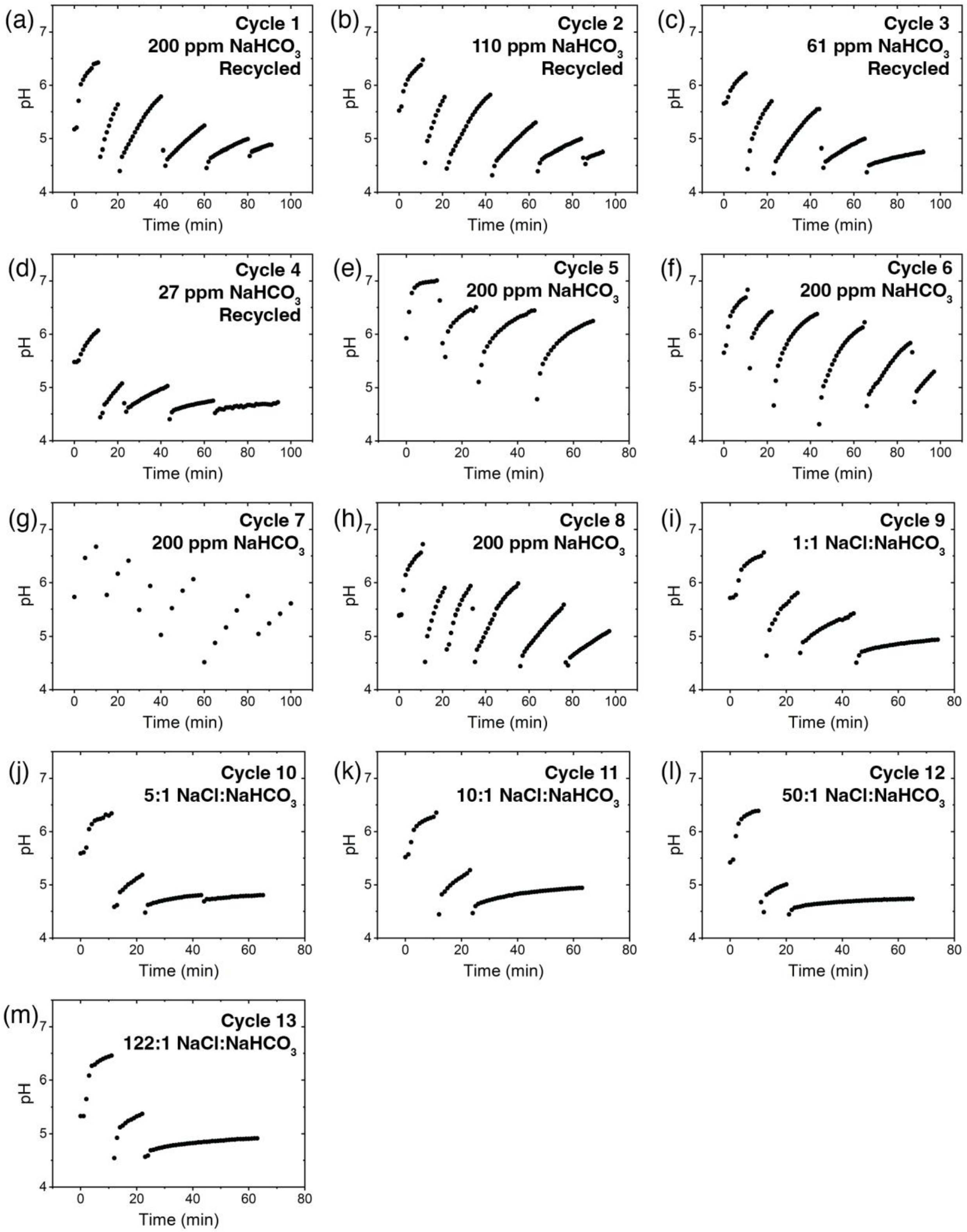

Figure S11: Solution pH versus time for all 13 regeneration cycles performed in this study.

Table S7: Changes in TIC for starting and ending solutions of cycles using recycled, 200 ppm $NaHCO_3$ solution.

Fibers	Cycle	Solution Composition	HCO_3^- (time 0 h) (ppm)	HCO_3^- (time 24 h) (ppm)	ΔHCO_3^- (ppm)	% Removal
**Control PVDF**		200 ppm $NaHCO_3$	134±0.4	129±3	--	--
**Guanidinium Functionalized PVDF**	**1**		133±1	87.3±1	-45.7±2	34
	**2**	200 ppm $NaHCO_3$ *Recycled*	79.9±0.3	48.5±1	-31.4±1	39
	**3**		44.1±1	20.2±2	-23.9±2	54
	**4**		19.3±0.1	3.2±1	-16.1±1	83

Table S8: Changes in TIC for starting and ending solutions of cycles using freshly prepared, 200 ppm $NaHCO_3$ solution.

Fibers	Cycle	Solution Composition	HCO_3^- (time 0 h) (ppm)	HCO_3^- (time 24 h) (ppm)	ΔHCO_3^- (ppm)	% Removal
**Guanidinium Functionalized PVDF**	**5**	200 ppm $NaHCO_3$	136±1	83.6±1	-52.4±2	39
	**6**		136±3	119±1	-17.0±3	12
	**7**		136±1	105±1	-31.0±2	23
	**8**		135±1	101±1	-34.0±2	25

Table S9: Changes in TIC for starting and ending solutions of cycles using freshly prepared solutions with varying concentrations of NaCl.

Fibers	Cycle	Solution Composition	HCO_3^- (time 0 h) (ppm)	HCO_3^- (time 24 h) (ppm)	ΔHCO_3^- (ppm)	% Removal
**Guanidinium Functionalized PVDF**	**9**	1:1 NaCl:$NaHCO_3$	136±2	114±1	-22.0±2	16
	**10**	5:1 NaCl:$NaHCO_3$	132±1	118±3	-14.0±3	11
	**11**	10:1 NaCl:$NaHCO_3$	135±1	120±2	-15.0±3	11
	**12**	50:1 NaCl:$NaHCO_3$	131±1	129±1	-2.0±2	2
	**13**	122:1 NaCl:$NaHCO_3$	137±0.4	134±2	-3.0±2	2

Quantum-Chemical Calculation

Optimized XYZ coordinates for all protonated amines and their bicarbonate (HCO_3^-) complexes.

Protonated amines				Protonated amine – bicarbonate (HCO_3^-) complexes			
**Methylamine**							
C	0.797663000000	0.000157000000	0.000083000000	C	0.470931140000	0.314033280000	0.823327420000
H	1.145360000000	0.492449000000	-0.904182000000	H	1.505594580000	0.374079200000	1.151383540000
H	1.144745000000	-1.029240000000	0.025626000000	H	-0.085180910000	-0.343028670000	1.486447960000
H	-1.048288000000	0.953021000000	-0.018968000000	H	1.047326670000	0.414401700000	-1.181261950000
N	-0.681656000000	0.000562000000	0.000161000000	N	0.429782190000	-0.188573420000	-0.560760360000
H	1.145778000000	0.537022000000	0.878463000000	H	0.045804810000	1.312635140000	0.864758270000
H	-1.047263000000	-0.492960000000	-0.814887000000	H	0.790908410000	-1.143617450000	-0.608306940000
H	-1.047770000000	-0.460597000000	0.833754000000	H	-0.520296970000	-0.161260010000	-0.936377660000
				H	4.326142500000	1.416953200000	-0.181638990000
				O	3.435599440000	1.151285940000	-0.459132500000
				O	4.018087510000	2.306588810000	-2.283812140000
				C	3.154178860000	1.639781730000	-1.719662800000
				O	2.024411760000	1.328201240000	-2.131469550000
**Dimethylamine**							
C	1.233975000000	-0.286330000000	0.000065000000	C	0.814284560000	-0.871651570000	-0.154489530000
H	1.237726000000	-0.906564000000	0.893646000000	H	1.007522690000	-0.973067590000	0.911257320000
H	2.096314000000	0.376268000000	-0.002432000000	H	1.741840900000	-0.653817770000	-0.676042990000
H	0.000226000000	1.137569000000	-0.819811000000	H	-0.358985570000	0.253537980000	-1.438182960000
N	-0.000003000000	0.528860000000	0.000277000000	N	-0.160143330000	0.200435560000	-0.393911590000
H	1.235144000000	-0.909862000000	-0.891242000000	H	0.413483740000	-1.806088270000	-0.540739240000
C	-1.233983000000	-0.286334000000	-0.000595000000	C	-1.425854070000	-0.028122580000	0.314471810000
H	-1.236556000000	-0.907131000000	-0.893791000000	H	-1.874881460000	-0.948695530000	-0.051398990000
H	-1.236324000000	-0.909298000000	0.891105000000	H	-1.247874060000	-0.112501310000	1.384394440000
H	-2.096318000000	0.376276000000	0.000351000000	H	-2.112930660000	0.790558610000	0.120947640000
H	-0.000209000000	1.136244000000	0.821359000000	H	0.236754620000	1.101231680000	-0.116030920000
				H	-1.635951120000	-2.547824140000	-3.134246400000
				O	-1.287246400000	-1.766270480000	-2.677536420000
				O	-1.430952180000	-0.911507350000	-4.739031590000
				C	-1.125600180000	-0.720569840000	-3.564834950000
				O	-0.676569760000	0.312173170000	-3.039231380000
**Triethylamine**							
C	-0.701999000000	1.224984000000	0.113528000000	C	-0.162898110000	0.518096220000	-0.664663350000
H	-0.710595000000	1.238123000000	1.202024000000	H	-0.447360370000	1.214726470000	0.122300450000
H	-1.719098000000	1.206347000000	-0.272249000000	H	-0.960733310000	0.454012670000	-1.399699210000
N	-0.001048000000	-0.000041000000	-0.331020000000	N	0.087443810000	-0.805721290000	-0.086296180000
H	-0.168301000000	2.091970000000	-0.270140000000	H	0.733554970000	0.877656260000	-1.162788300000
C	-0.708719000000	-1.221244000000	0.113520000000	C	-1.122525400000	-1.348055230000	0.540252810000

H	-0.179381000000	-2.091158000000	-0.269561000000	H	-0.924453290000	-2.357838500000	0.891650880000
H	-0.718033000000	-1.234007000000	1.202011000000	H	-1.419331630000	-0.718426190000	1.377146880000
H	-1.725466000000	-1.197344000000	-0.272923000000	H	-1.928335480000	-1.389460520000	-0.187902030000
C	1.410498000000	-0.003749000000	0.112182000000	C	1.196036100000	-0.764350510000	0.872998770000
H	1.901050000000	0.888008000000	-0.272035000000	H	2.088323860000	-0.375888320000	0.388975740000
H	1.426552000000	-0.004128000000	1.200692000000	H	0.931580960000	-0.128457490000	1.716050920000
H	1.896575000000	-0.897713000000	-0.272581000000	H	1.406167770000	-1.771560340000	1.224992310000
H	-0.002525000000	-0.000051000000	-1.353221000000	H	0.367442980000	-1.463500290000	-0.869875660000
				H	0.996931680000	-4.836210530000	-0.207007030000
				O	0.829384400000	-3.901707370000	-0.406987230000
				O	1.371012420000	-4.602160010000	-2.460464060000
				C	1.026250320000	-3.658546050000	-1.754075290000
				O	0.821878440000	-2.478228550000	-2.081504040000

Triethylenediamine

C	0.330835000000	1.536698000000	0.042992000000	C	-0.239477390000	1.672399060000	0.870027930000
C	1.483353000000	0.519073000000	0.039986000000	C	0.174180140000	1.691134620000	-0.619538070000
N	0.877811000000	-0.840033000000	-0.027460000000	N	-0.268367440000	0.419801380000	-1.222485330000
C	0.062682000000	-0.975645000000	-1.266448000000	C	-1.734260830000	0.286424180000	-1.115146240000
C	-1.068260000000	0.063043000000	-1.189900000000	C	-2.098657420000	0.276732460000	0.386177920000
N	-0.968685000000	0.869764000000	0.023647000000	N	-0.900164210000	0.421013150000	1.191076010000
H	0.400242000000	2.183776000000	-0.831055000000	H	-0.927512750000	2.487519790000	1.095572340000
H	0.389094000000	2.162337000000	0.933191000000	H	0.630893440000	1.774270290000	1.518705250000
H	2.083062000000	0.556658000000	0.947840000000	H	1.253614160000	1.770814240000	-0.745177640000
H	2.135992000000	0.621942000000	-0.825451000000	H	-0.293384020000	2.509861140000	-1.165703710000
H	-0.306053000000	-1.999457000000	-1.297359000000	H	-2.024834410000	-0.635130280000	-1.619772060000
H	0.732380000000	-0.809944000000	-2.108764000000	H	-2.191533180000	1.123451590000	-1.642649150000
H	-1.024045000000	0.726067000000	-2.053525000000	H	-2.773996880000	1.097478020000	0.629310860000
H	-2.036437000000	-0.436999000000	-1.196052000000	H	-2.586152130000	-0.657803980000	0.664756150000
C	0.029784000000	-1.084405000000	1.172089000000	C	0.394196120000	-0.718973170000	-0.557473050000
H	0.681957000000	-1.033406000000	2.042354000000	H	1.470141010000	-0.617230200000	-0.698495460000
H	-0.368704000000	-2.093567000000	1.080954000000	H	0.069217540000	-1.632503330000	-1.055678360000
C	-1.068530000000	-0.008448000000	1.186315000000	C	-0.001860720000	-0.689193780000	0.935602370000
H	-2.050718000000	-0.480438000000	1.184567000000	H	-0.507031840000	-1.611217130000	1.224323570000
H	-0.986557000000	0.598718000000	2.087491000000	H	0.876376580000	-0.570317590000	1.570718900000
H	1.625755000000	-1.532258000000	-0.049400000000	H	-0.002340530000	0.411025380000	-2.251387280000
				H	-0.839900640000	-2.302459520000	-4.216355040000
				O	-0.594465870000	-1.535620480000	-3.673338130000
				O	0.247944010000	-0.850484260000	-5.627766990000
				C	0.068985250000	-0.598562140000	-4.441158130000
				O	0.409752420000	0.414857840000	-3.804465490000

Piperidine

C	-0.750582000000	-1.250234000000	-0.228415000000	C	-0.985148820000	-0.909817930000	-0.445988170000
C	-1.490493000000	-0.000489000000	0.223711000000	C	-1.546325590000	0.124603280000	0.526068690000

C	-0.751340000000	1.249761000000	-0.228233000000	C	-0.759596890000	1.425670110000	0.393020890000
C	0.683764000000	1.241094000000	0.243781000000	C	0.718267390000	1.166112630000	0.648807320000
N	1.365785000000	0.000359000000	-0.222912000000	N	1.203007570000	0.168594850000	-0.323614980000
C	0.684414000000	-1.240708000000	0.243834000000	C	0.500150500000	-1.121427300000	-0.186767100000
H	-0.767284000000	-1.320469000000	-1.321572000000	H	-1.118173020000	-0.556268380000	-1.470832440000
H	-1.227332000000	-2.152572000000	0.159797000000	H	-1.512372440000	-1.857669500000	-0.344848110000
H	-1.571037000000	-0.000607000000	1.316228000000	H	-1.471205010000	-0.250862730000	1.548101780000
H	-2.506885000000	-0.000777000000	-0.175524000000	H	-2.599346980000	0.302660240000	0.310269350000
H	-0.768252000000	1.320246000000	-1.321364000000	H	-0.877247570000	1.822342310000	-0.617429340000
H	-1.228507000000	2.151755000000	0.160272000000	H	-1.129054820000	2.169703720000	1.097205450000
H	0.749790000000	1.237141000000	1.332681000000	H	0.865239630000	0.775592720000	1.658016720000
H	1.251711000000	2.086678000000	-0.141283000000	H	1.289077110000	2.086019620000	0.527283590000
H	1.390394000000	0.000403000000	-1.245422000000	H	1.026749220000	0.538953440000	-1.307142500000
H	2.333056000000	0.000641000000	0.102394000000	H	2.210729400000	0.030732370000	-0.231453350000
H	0.750237000000	-1.236473000000	1.332752000000	H	0.658368560000	-1.505573530000	0.822957870000
H	1.253060000000	-2.086001000000	-0.140837000000	H	0.914022910000	-1.819425340000	-0.914705550000
				H	2.199876530000	-1.253603630000	-4.008208040000
				O	1.851557570000	-0.735336380000	-3.265875650000
				O	1.176988200000	0.588915970000	-4.939255450000
				C	1.216188830000	0.403338770000	-3.726210070000
				O	0.750405690000	1.110005180000	-2.817412420000

Pyridine

C	-1.416257000000	-0.000004000000	-0.000009000000	C	-1.383390860000	-1.143748600000	0.343850070000
C	-0.722711000000	1.202056000000	-0.000010000000	C	-2.080487520000	0.054494060000	0.431597400000
C	-0.722705000000	-1.202062000000	-0.000009000000	C	-0.037100050000	-1.124376820000	0.000912060000
H	-1.237211000000	2.152722000000	-0.000010000000	H	-3.124632450000	0.073822860000	0.694873890000
H	-1.237199000000	-2.152731000000	-0.000004000000	H	0.530559110000	-2.036573260000	-0.075091930000
C	0.651287000000	1.176881000000	-0.000010000000	C	-1.411290330000	1.236432880000	0.173833940000
C	0.651294000000	-1.176877000000	-0.000010000000	C	0.572443950000	0.091795180000	-0.244304130000
H	1.272006000000	2.061381000000	-0.000006000000	H	-1.883469900000	2.206197250000	0.221915820000
H	1.272018000000	-2.061374000000	-0.000011000000	H	1.613034830000	0.179867110000	-0.514554650000
N	1.286323000000	0.000004000000	-0.000009000000	N	-0.122235480000	1.222604150000	-0.152303560000
H	-2.499226000000	-0.000006000000	-0.000008000000	H	-1.882228490000	-2.079511750000	0.539989240000
H	2.301876000000	0.000008000000	-0.000010000000	H	0.363296780000	2.142818990000	-0.343197580000
				H	-1.303920570000	5.227740680000	-0.100284930000
				O	-0.839162400000	4.378317520000	-0.171955570000
				O	0.852747740000	5.742957380000	-0.692839180000
				C	0.480068880000	4.585139490000	-0.527880650000
				O	1.138202400000	3.536890870000	-0.637789260000

Guanidine

C	0.000001000000	0.000226000000	0.000004000000	C	-0.133415880000	0.023179840000	0.004212640000
N	1.037768000000	0.823698000000	0.000004000000	N	0.930551160000	0.789920260000	-0.183093390000
N	-1.231752000000	0.487555000000	0.000000000000	N	-1.351808300000	0.568367550000	0.007004750000
N	0.193461000000	-1.310583000000	0.000005000000	N	0.005458860000	-1.282324700000	0.178741420000
H	0.898304000000	1.820597000000	-0.000013000000	H	0.837574630000	1.782574730000	-0.305246600000

H	-2.024717000000	-0.132789000000	-0.000009000000	H	-2.167530310000	-0.006456510000	0.120558140000
H	-1.386004000000	1.482216000000	0.000006000000	H	-1.471238370000	1.552043900000	-0.156403770000
H	1.126916000000	-1.687488000000	-0.000009000000	H	0.932378310000	-1.707589550000	0.210012810000
H	-0.591322000000	-1.941174000000	0.000001000000	H	-0.791911900000	-1.869882860000	0.345412680000
H	1.976352000000	0.459846000000	0.000004000000	H	1.869372380000	0.391239990000	-0.157074650000
				H	5.504562740000	-1.496206900000	0.072428950000
				O	4.819466280000	-2.174337540000	0.203227500000
				O	3.518006380000	-0.383860720000	-0.061744600000
				C	3.573749390000	-1.605958030000	0.138563390000
				O	2.621014480000	-2.377136250000	0.288679880000
**1-methylguanidine**							
C	-0.478533000000	0.020834000000	-0.000216000000	C	-0.640419620000	-0.180013420000	0.046189490000
N	0.686215000000	0.651372000000	-0.000554000000	N	0.389283260000	0.512344450000	-0.417406590000
N	-1.609726000000	0.717804000000	0.000166000000	N	-1.857671750000	0.352183260000	0.021088320000
N	-0.522246000000	-1.304095000000	-0.000409000000	N	-0.468121020000	-1.410313370000	0.535687510000
H	0.670349000000	1.659074000000	0.001152000000	H	0.219971880000	1.450446470000	-0.784342260000
H	-2.498109000000	0.244796000000	0.000280000000	H	-2.648402010000	-0.153810800000	0.377720570000
H	-1.588315000000	1.724079000000	0.000085000000	H	-2.007591010000	1.290266140000	-0.347710790000
H	-1.407504000000	-1.783272000000	-0.000639000000	H	-1.251167900000	-1.931705140000	0.886710470000
H	0.323242000000	-1.849308000000	-0.000410000000	H	0.446686630000	-1.822968420000	0.563055180000
C	1.963223000000	-0.025430000000	0.000117000000	C	1.751102550000	0.023826070000	-0.441337290000
H	2.084058000000	-0.646693000000	-0.890226000000	H	1.834509200000	-0.874343170000	-1.055594270000
H	2.743181000000	0.731457000000	-0.001432000000	H	2.373206400000	0.799021550000	-0.877397090000
H	2.084765000000	-0.644129000000	0.892163000000	H	2.111428780000	-0.182321890000	0.568163660000
				H	-2.261457430000	5.080710570000	-1.915009170000
				O	-1.324859330000	4.822159340000	-1.960976040000
				O	-2.169465720000	2.980756090000	-1.030828600000
				C	-1.157486870000	3.562235710000	-1.448544670000
				O	-0.001648580000	3.127161490000	-1.454029090000
**1,1-Dimethylguanidine**							
C	-0.661181000000	-0.001457000000	0.000744000000	C	-0.612766500000	0.109739190000	-0.144070020000
N	0.664277000000	0.001842000000	0.000769000000	N	0.704197590000	0.024434210000	0.063993440000
N	-1.343893000000	1.143192000000	0.013183000000	N	-1.194652880000	1.298792150000	-0.287643570000
N	-1.338106000000	-1.149291000000	-0.010949000000	N	-1.359369710000	-0.991201910000	-0.205216630000
H	-0.884596000000	2.016837000000	-0.179855000000	H	-0.720956440000	2.141338000000	-0.015769180000
H	-2.347092000000	1.111620000000	-0.072863000000	H	-2.203876200000	1.352293510000	-0.426414860000
H	-2.341716000000	-1.123493000000	0.071770000000	H	-2.357703370000	-0.911480980000	-0.401486180000
H	-0.874387000000	-2.021598000000	0.177346000000	H	-0.938298450000	-1.892809430000	-0.340794610000
C	1.383863000000	1.263176000000	0.036539000000	C	1.548113450000	1.200481570000	-0.015995390000
H	1.092741000000	1.854552000000	0.907640000000	H	1.498172100000	1.790779700000	0.901379080000
H	2.446629000000	1.052233000000	0.108515000000	H	2.575111890000	0.882379180000	-0.169236370000
H	1.209278000000	1.846656000000	-0.871470000000	H	1.258122890000	1.819235440000	-0.863916450000
C	1.389966000000	-1.255922000000	-0.036737000000	C	1.333583770000	-1.247636890000	0.358581760000
H	2.451803000000	-1.039744000000	-0.106675000000	H	1.497873310000	-1.832023780000	-0.549202440000
H	1.216981000000	-1.842066000000	0.869850000000	H	2.293827240000	-1.059720440000	0.830057330000

H	1.102952000000	-1.846921000000	-0.909480000000	H	0.724508510000	-1.821104420000	1.055206270000
				H	-6.254676600000	1.374588900000	-1.106621720000
				O	-5.961634890000	0.447032590000	-1.092016470000
				O	-4.015479140000	1.451043010000	-0.674100590000
				C	-4.616772520000	0.379321810000	-0.837459780000
				O	-4.131709950000	-0.755744840000	-0.796404090000

1,1,3-Trimethylguanidine

C	-0.259258000000	-0.625493000000	0.120094000000	C	-0.587750080000	0.012505950000	0.298827190000
N	0.802889000000	0.145539000000	-0.097428000000	N	0.670232760000	0.079412060000	-0.150896330000
N	-0.108281000000	-1.945477000000	0.223997000000	N	-1.043118090000	-1.117667780000	0.838017220000
N	-1.489900000000	-0.118664000000	0.227375000000	N	-1.423005030000	1.053876310000	0.214667040000
H	0.783728000000	-2.344600000000	0.463683000000	H	-0.412035850000	-1.822524200000	1.177338830000
H	-0.920773000000	-2.540233000000	0.240997000000	H	-2.015829480000	-1.176220380000	1.136600420000
H	-2.194269000000	-0.769393000000	0.539645000000	H	-2.318220140000	0.924736880000	0.673894720000
C	0.853513000000	1.531943000000	0.334725000000	C	1.423249000000	1.316291530000	-0.222750420000
H	0.013843000000	1.766269000000	0.982653000000	H	0.868483330000	2.129366980000	0.231064480000
H	1.770653000000	1.675036000000	0.909073000000	H	2.353590520000	1.187004240000	0.331512140000
H	0.862866000000	2.216515000000	-0.515107000000	H	1.667484620000	1.567189680000	-1.255300420000
C	2.075347000000	-0.438658000000	-0.476377000000	C	1.469270960000	-1.121449330000	-0.289116850000
H	2.647975000000	-0.762536000000	0.398103000000	H	1.956889190000	-1.396168290000	0.649254830000
H	1.924632000000	-1.282996000000	-1.147431000000	H	0.852499450000	-1.946676060000	-0.636470420000
H	2.650697000000	0.319442000000	-1.006356000000	H	2.237818300000	-0.930027200000	-1.034923970000
C	-1.973396000000	1.062302000000	-0.463869000000	C	-1.436963490000	2.051174570000	-0.840503480000
H	-2.060374000000	1.921520000000	0.202262000000	H	-2.434116060000	2.083077490000	-1.276990550000
H	-1.315313000000	1.317352000000	-1.292177000000	H	-1.208212900000	3.040214820000	-0.443014390000
H	-2.958933000000	0.836935000000	-0.869537000000	H	-0.726535690000	1.792924060000	-1.619570960000
				H	-4.958593930000	1.630984380000	1.405533350000
				O	-4.240849390000	0.987690090000	1.288115650000
				O	-5.775159010000	-0.356270220000	2.202100780000
				C	-4.631544810000	-0.262108380000	1.760857320000
				O	-3.753374050000	-1.125344160000	1.670592400000

1,1,3,3-Tetramethylguanidine

C	0.000889000000	-0.464354000000	-0.148703000000	C	0.413284000000	-0.513273000000	-0.368909000000
N	1.185913000000	0.148139000000	-0.058763000000	N	1.416643000000	0.132629000000	0.240666000000
N	-0.039871000000	-1.756216000000	-0.463561000000	N	0.607412000000	-1.743003000000	-0.828547000000
N	-1.143853000000	0.190789000000	0.069230000000	N	-0.790015000000	0.062416000000	-0.495569000000
H	0.787805000000	-2.325566000000	-0.411840000000	H	1.436972000000	-2.240070000000	-0.550675000000
H	-0.898585000000	-2.188225000000	-0.759450000000	H	0.047549000000	-2.137312000000	-1.598581000000
C	1.424162000000	1.235999000000	0.873855000000	C	1.192638000000	1.079655000000	1.316894000000
H	1.705974000000	2.151927000000	0.351262000000	H	1.504158000000	2.087629000000	1.035737000000
H	0.545626000000	1.425585000000	1.483562000000	H	0.145797000000	1.095559000000	1.607013000000
H	2.242082000000	0.947887000000	1.538608000000	H	1.779828000000	0.764890000000	2.182970000000
C	2.380908000000	-0.490464000000	-0.575981000000	C	2.786117000000	-0.317995000000	0.094555000000
H	3.124045000000	0.284039000000	-0.762212000000	H	3.443155000000	0.543071000000	0.218722000000
H	2.799828000000	-1.209080000000	0.136065000000	H	3.051244000000	-1.066817000000	0.848149000000

H	2.170039000000	-0.991770000000	-1.519915000000	H	2.952098000000	-0.728609000000	-0.900332000000
C	-2.380955000000	-0.540470000000	0.263788000000	C	-1.965419000000	-0.720003000000	-0.819883000000
H	-3.073696000000	0.104334000000	0.803428000000	H	-2.827184000000	-0.251918000000	-0.341397000000
H	-2.841290000000	-0.823502000000	-0.688536000000	H	-2.144375000000	-0.754125000000	-1.898188000000
H	-2.208364000000	-1.432017000000	0.865421000000	H	-1.867899000000	-1.733411000000	-0.435997000000
C	-1.302454000000	1.598449000000	-0.251381000000	C	-0.947535000000	1.491959000000	-0.687439000000
H	-1.518947000000	2.188314000000	0.641234000000	H	-1.539671000000	1.938518000000	0.113800000000
H	-0.411966000000	1.990377000000	-0.734530000000	H	0.017120000000	1.988079000000	-0.740511000000
H	-2.137914000000	1.701221000000	-0.947927000000	H	-1.466205000000	1.658274000000	-1.635301000000
				H	-1.886617000000	-3.417133000000	-4.823327000000
				O	-1.481764000000	-2.570516000000	-5.054216000000
				O	-0.711678000000	-2.953970000000	-2.984727000000
				C	-0.740297000000	-2.167384000000	-3.960697000000
				O	-0.183551000000	-1.064945000000	-4.078462000000

Example of input files

Optimization and frequencies

```
! opt freq
! PBE0
! RHF
! D3BJ
! def2-TZVPD def2-TZVPD/C def2/J def2/JK
! defgrid3
! PModel
! SMD(water)
%scf
  CNVSOSCF TRUE
  SCFmode  Direct
  MaxIter  800
```

```
end
%geom
  MaxIter 250
  coordsys redundant
  inhess   Almloef
end
%pal
  nprocs 16
  nprocs_group 4
end
* xyz 1 1
```
coordinates

```
*
```

Docking

```
! XTB PAL16 ALPB(WATER) NORMALDOCK
%pal
  nprocs 16
  nprocs_group 4
end
%DOCKER
  GUEST "HCO3.xyz"
  GUESTCHARGE -1
  GUESTMULT   1
```

DOCKLEVEL NORMAL

END

* xyz 1 1

coordinates

*

References

(1) Bader, R. F. W. Atoms in molecules. *Accounts of Chemical Research* **1985**, *18* (1), 9-15. DOI: 10.1021/ar00109a003.

(2) Pracht, P.; Grimme, S.; Bannwarth, C.; Bohle, F.; Ehlert, S.; Feldmann, G.; Gorges, J.; Müller, M.; Neudecker, T.; Plett, C.; et al. CREST-A program for the exploration of low-energy molecular chemical space. *J Chem Phys* **2024**, *160* (11). DOI: 10.1063/5.0197592 From NLM.

(3) Bannwarth, C.; Ehlert, S.; Grimme, S. GFN2-xTB—An Accurate and Broadly Parametrized Self-Consistent Tight-Binding Quantum Chemical Method with Multipole Electrostatics and Density-Dependent Dispersion Contributions. *Journal of Chemical Theory and Computation* **2019**, *15* (3), 1652-1671. DOI: 10.1021/acs.jctc.8b01176.

(4) Neese, F. Software Update: The ORCA Program System—Version 6.0. *WIREs Computational Molecular Science* **2025**, *15* (2), e70019. DOI: https://doi.org/10.1002/wcms.70019.

(5) Adamo, C.; Barone, V. Toward reliable density functional methods without adjustable parameters: The PBE0 model. *The Journal of Chemical Physics* **1999**, *110* (13), 6158-6170. DOI: 10.1063/1.478522 (acccessed 10/20/2025).

(6) Grimme, S.; Antony, J.; Ehrlich, S.; Krieg, H. A consistent and accurate ab initio parametrization of density functional dispersion correction (DFT-D) for the 94 elements H-Pu. *The Journal of Chemical Physics* **2010**, *132* (15). DOI: 10.1063/1.3382344 (acccessed 10/20/2025).

(7) Grimme, S.; Ehrlich, S.; Goerigk, L. Effect of the damping function in dispersion corrected density functional theory. *Journal of Computational Chemistry* **2011**, *32* (7), 1456-1465. DOI: https://doi.org/10.1002/jcc.21759.

(8) Rappoport, D.; Furche, F. Property-optimized gaussian basis sets for molecular response calculations. *J Chem Phys* **2010**, *133* (13), 134105. DOI: 10.1063/1.3484283 From NLM.

(9) Weigend, F.; Ahlrichs, R. Balanced basis sets of split valence, triple zeta valence and quadruple zeta valence quality for H to Rn: Design and assessment of accuracy. *Physical Chemistry Chemical Physics* **2005**, *7* (18), 3297-3305, 10.1039/B508541A. DOI: 10.1039/B508541A.

(10) Marenich, A. V.; Cramer, C. J.; Truhlar, D. G. Universal Solvation Model Based on Solute Electron Density and on a Continuum Model of the Solvent Defined by the Bulk Dielectric Constant and Atomic Surface Tensions. *The Journal of Physical Chemistry B* **2009**, *113* (18), 6378-6396. DOI: 10.1021/jp810292n.

(11) Bannwarth, C.; Caldeweyher, E.; Ehlert, S.; Hansen, A.; Pracht, P.; Seibert, J.; Spicher, S.; Grimme, S. Extended tight-binding quantum chemistry methods. *WIREs Computational Molecular Science* **2021**, *11* (2), e1493. DOI: https://doi.org/10.1002/wcms.1493.

(12) Lu, T.; Chen, F. Multiwfn: A multifunctional wavefunction analyzer. *Journal of Computational Chemistry* **2012**, *33* (5), 580-592. DOI: https://doi.org/10.1002/jcc.22885.

(13) Baldwin, D. A.; Denner, L.; Egan, T. J.; Markwell, A. J. Structure of guanidinium bicarbonate: a model for the bicarbonate anion binding site of the transferrins. *Acta Crystallographica Section C* **1986**, *42* (9), 1197-1199. DOI: doi:10.1107/S0108270186092909.

(14) Roy, A.; Holmes, H. E.; Baugh, L. S.; Calabro, D. C.; Leisen, J.; Seth, S.; Ren, Y.; Weston, S. C.; Lively, R. P.; Finn, M. G. Guanidine-Functionalized PIM-1 as a High-Capacity Polymeric Sorbent for CO2 Capture. *Chemistry of Materials* **2024**, *36* (9), 4393-4402. DOI: 10.1021/acs.chemmater.3c03311.
